# The Collaborative Collapse: Bile Acid Dysmetabolism as a Central Pathogenic Driver in Canine and Feline Multi-Systemic Disorders—From Mechanisms to Precision Therapeutics

**DOI:** 10.3390/vetsci13020182

**Published:** 2026-02-12

**Authors:** Krisztián Németh, István Tóth, Katalin Lányi, Boglárka Mária Schilling-Tóth, Szilveszter Csorba, Ivona Žura Žaja, Ágnes Sterczer

**Affiliations:** 1 Department of Physiology and Biochemistry, University of Veterinary Medicine Budapest, István u. 2, H-1078 Budapest, Hungary; nemeth.krisztian@univet.hu (K.N.); schilling-toth.boglarka.maria@univet.hu (B.M.S.-T.); 2 Food Chain Laboratory Center, Institute of Food Chain Science, University of Veterinary Medicine Budapest, Marek J. u. 2, H-1078 Budapest, Hungary; lanyi.katalin@univet.hu; 3 Department of Digital Food Science, Institute of Food Chain Science, University of Veterinary Medicine Budapest, István u. 2, H-1078 Budapest, Hungary; csorba.szilveszter@univet.hu; 4 Department of Physiology and Radiobiology, Faculty of Veterinary Medicine, University of Zagreb, Heinzelova 55, 10000 Zagreb, Croatia; izzaja@vef.unizg.hr; 5 Department of Internal Medicine, University of Veterinary Medicine Budapest, István u. 2, H-1078 Budapest, Hungary; sterczer.agnes@univet.hu

**Keywords:** bile acids, microbiome, *Peptacetobacter hiranonis*, chronic enteropathy, TGR5, MCBA, Gut–X axis, SynComs, extracellular vesicles, postbiotics

## Abstract

Veterinary metabolomics has redefined bile acids (BAs) from simple digestive surfactants to systemic endocrine signals within a microbial–host metabolic axis. This review aims to evaluate how BA dysmetabolism acts as a central pathogenic factor in canine and feline disease. We analyze the BA pool’s integrity, which depends on a specialized functional guild, primarily *Peptacetobacter hiranonis*, responsible for 7α-dehydroxylation. We delineate two principal pathological profiles: (1) microbial collapse, characterized by secondary bile acid (SBA) depletion and compromised farnesoid X receptor (FXR) and Takeda G protein-coupled receptor 5 (TGR5) signaling, which exacerbates inflammation in chronic enteropathy (CE), protein-losing enteropathy (PLE), and exocrine pancreatic insufficiency (EPI); and (2) hepato-biliary spillover, wherein host-induced dysfunction results in primary bile acid (PBA) excess. Recent data have linked these disruptions to skeletal health, feline renal fibrosis, cardiac remodeling in myxomatous mitral valve disease (MMVD), and neuroinflammation in epilepsy and hepatic encephalopathy. The discovery of microbially conjugated bile acids (MCBAs) and microbial extracellular vesicles (MEVs) reveals highly specific, vesicle-mediated communication pathways impacting systemic health. Diagnostic protocols should prioritize functional profiling, including the dysbiosis index (DI), serum conjugated BA analysis, and SBA/PBA ratios. Clinical management is moving beyond empirical fecal microbiota transplantation (FMT), towards precision synthetic microbial consortia (SynComs), neuroprotective BAs like tauroursodeoxycholic acid (TUDCA), and molecular postbiotics to restore the collaborative metabolome.

## Simple Summary

Bile acids are best known for their role in digestion, but they also act as vital chemical signals that keep an animal’s metabolism, immune system, and organs healthy. In dogs and cats, this robust balance is often disrupted in two specific ways. Sometimes, the beneficial gut bacteria that process these acids are lost due to antibiotics or illness, leading to a “microbial collapse” that is often associated with chronic diarrhea or can remain asymptomatic. In other cases, liver issues cause toxic bile acids to “spill over” into the digestive tract. Our review shows that these two problems are distinct and require very different approaches: one needs a restoration of healthy bacteria, while the other requires medication to trap and remove excess acids. By identifying these specific patterns, veterinarians can move away from trial-and-error treatments and use more targeted therapies. This shift toward precision medicine helps ensure that companion animals suffering from complex, long-term digestive and liver diseases can lead more comfortable and healthy lives.

## 1. Introduction: The Modern Reconstruction of Bile Acid Physiology

### 1.1. From Digestive Detergents to Pleiotropic Signaling Molecules

For nearly a century, the physiological role of bile acids (BAs) in veterinary medicine was interpreted through a limited perspective. As amphipathic molecules derived from cholesterol catabolism, they were recognized primarily as surfactants needed for the emulsification of dietary lipids and the absorption of fat-soluble vitamins in the proximal small intestine [1,2]. This traditional focus on the detergent properties of BAs, while physiologically accurate, overshadowed their far-reaching regulatory potential. The field changed markedly at the turn of the 21st century with the identification of dedicated BA receptors, specifically the nuclear FXR and the membrane-bound Takeda G protein-coupled (bile acid) receptor 5 (TGR5) (encoded by the GPBAR1 gene) [3,4,5].

These discoveries redefined our understanding of the BA pool, repositioning it as a systemic, hormone-like signaling network. We now recognize that BAs function as metabolic master regulators, coordinating complex pathways in energy expenditure, glucose homeostasis, and hepatic lipid metabolism [6,7]. Beyond metabolism, they act as potent immunomodulators, shaping mucosal immune tolerance and suppressing systemic inflammatory responses through the activation of TGR5 on resident macrophages and dendritic cells [8,9]. In the modern veterinary context, BAs must be viewed as central participants in the cross-talk between the gut and the entire organism, distinct from their role as simple metabolic end-products [10].

This molecular dialog extends beyond soluble metabolites. Evidence identifies microbial extracellular vesicles (MEVs) as lipid-bilayer-enclosed “packets” that facilitate long-distance communication between the microbe and the host. These nano-sized vesicles ensure the protected transport of enzymes, RNA, and metabolites across the epithelial barrier. By entering the portal circulation, these vesicles exert a direct influence on hepatic responses that free metabolites cannot achieve [11]. Alongside BAs, they serve as essential vectors of this symbiotic network [12]. This communication is reciprocal. For instance, BAs induce the production of immunomodulatory vesicles by commensals such as *Lactobacillus johnsonii*. This interaction establishes a feedback mechanism in which the host’s metabolic state regulates microbial signaling output [13].

### 1.2. The Endocrine Network and the Collaborative Metabolome

The functional BA pool is not produced by a single organ but is the output of a collaborative metabolome [14,15]. This concept highlights the interaction between the host and the gut microbiome. While the liver synthesizes primary bile acids (PBAs)—cholic acid (CA) and chenodeoxycholic acid (CDCA)—microbial modification in the distal gut is essential to reach the full signaling diversity of the pool [16].

In dogs and cats, this relationship is specific. Cats are strict obligate taurine conjugators, while dogs preferentially conjugate with taurine (approx. 99%) but retain a limited capacity for glycine conjugation. This makes their enterohepatic circulation sensitive to dietary amino acid (AA) availability and microbial deconjugation [17,18]. The synthesis of anti-inflammatory secondary bile acids (SBAs), such as deoxycholic acid (DCA) and lithocholic acid (LCA), depends on a specialized guild of bacteria carrying the *bai* (bile acid-inducible) operon [19,20,21]. In the canine microbiome, this guild is dominated by *Peptacetobacter* (formerly *Clostridium*) *hiranonis*, whose presence dictates the metabolic health of the intestinal environment [22,23,24].

### 1.3. The Pathological Dichotomy: Collapse vs. Spillover

The fragility of this host–microbe functional relationship represents a significant biological vulnerability. When the collaboration fails, the resulting BA dysmetabolism drives disease progression. This review proposes a classification to categorize these metabolic defects, moving beyond the nonspecific clinical label of dysbiosis.

Microbial collapse (functional failure) is defined by the eradication of the 7α-dehydroxylating guild (e.g., post-antibiotic or substrate-driven loss of *P. hiranonis*), resulting in a substantial reduction in anti-inflammatory SBAs. This leads to “TGR5 signaling starvation”, a condition that promotes unchecked mucosal inflammation and diminished secretion of glucagon-like peptide-1 (GLP-1), now confirmed as a fundamental characteristic of chronic enteropathy (CE), protein-losing enteropathy (PLE), and exocrine pancreatic insufficiency (EPI) [25,26,27].

Hepatobiliary spillover (containment failure) is characterized by an uncontrolled influx of primary BAs into the lower intestine resulting from host-induced hepatobiliary dysfunction, cholestasis, or failure of the apical sodium-dependent bile acid transporter (ASBT). This profile encompasses two distinct categories: colonic spillover, which induces secretory diarrhea, and systemic spillover (vascular bypass), wherein congenital or acquired portosystemic shunts (PSSs) allow diminished portal bile acids to bypass hepatic extraction, resulting in systemic neurotoxicity and “signaling chaos” [28,29]. The surplus of PBAs induces secretory bile acid diarrhea (BAD) via osmotic and detergent-like toxicity to the intestinal mucosa, frequently irrespective of microbial condition [30,31,32,33].

### 1.4. Beyond the Gut–Liver Axis: The “Gut–X” Paradigm

The systemic distribution of BAs and their corresponding receptors implies that the consequences of dysmetabolism extend to nearly every organ system. This observation aligns with the broader biological principle of organ crosstalk, where molecular signaling between distant tissues maintains physiological homeostasis [34]. High-resolution profiling has identified BA signaling hubs in the heart, kidneys, and central nervous system, establishing a “gut–X” network [35,36]. The clinical impact of these microbial shifts is best understood through a series of systemic axes.

The gut–immune axis illustrates how a deficiency in SBAs triggers chronic mucosal inflammation, largely driven by maladaptive macrophage polarization. This dysregulation extends to the gut–heart axis, where altered bile acid profiles (BAPs) and the accumulation of pro-fibrotic metabolites, such as trimethylamine N-oxide (TMAO), correlate with the progression of advanced myxomatous mitral valve disease (MMVD) in dogs [37,38]. MEVs further contribute to myocardial remodeling by translocating to the heart to modulate inflammatory pathways independently of TMAO [39].

The gut–kidney axis follows a related pattern, with the loss of cytoprotective SBAs like ursodeoxycholic acid (UDCA), a shift closely linked to the advancement of renal fibrosis in feline chronic kidney disease (CKD) [40,41]. Beyond internal organs, research is increasingly defining a gut–skin axis, connecting microbial metabolite deficiencies to atopic dermatitis (AD)—and broader cutaneous immunity—and a gut–joint axis, where dysbiosis and barrier permeability drive osteoarthritis through metabolite-mediated articular inflammation. Emerging preclinical evidence suggests this systemic influence reaches the gut–bone axis, where dysbiosis and increased permeability disrupt osteoimmune regulation. This process is increasingly linked to the translocation of bacterial extracellular vesicles—delivering bioactive signals directly to osteoblasts—and a complex brain–gut–bone circuit involving serotonin signaling [42,43,44]. The concept further extends to the gut–lung, gut–reproductive, and gut–endocrine axes, reflecting the microbiome’s broad role in homeostasis [45,46,47,48,49,50].

Perhaps the most complex extension is the gut–brain axis. BAs act as neuroactive steroids that cross the blood–brain barrier (BBB) to modulate neuroinflammation and synaptic plasticity through regional signaling in the cortex and hippocampus [51,52]. The disruption of this signaling—marked by alterations in brain-derived neurotrophic factor (BDNF) pathways—is increasingly implicated in canine epilepsy, hepatic encephalopathy (HE), and cognitive dysfunction syndrome (CDS) [53,54]. This communication is further supported by MEVs, which traverse the BBB to deliver neuroactive cargo directly to glial cells [55].

Recognizing these systemic links is essential for transitioning veterinary medicine from organ-centric to axis-centric clinical care.

### 1.5. Aims and Scope of the Review

Current veterinary literature frequently addresses BAs in a fragmented manner, viewing them either as static markers of liver function or as incidental findings in gastrointestinal (GI) disease. This review synthesizes the available evidence to reconstruct BA physiology as an integrated endocrine system. We aim to examine the molecular architecture of the collaborative metabolome and the role of the *bai* operon guild in maintaining host homeostasis. By characterizing the pathophysiology of signaling failure, we establish a clear clinical distinction between the “collapse” and “spillover” profiles, providing a necessary foundation for mechanism-based diagnostics. This analysis extends to the systemic implications of BA dysmetabolism, mapping its influence across the broadening “gut–X” axes to clarify its role in renal, cardiovascular, neurological, musculoskeletal, and cutaneous health. The review closes by emphasizing the importance of incorporating fecal metabolic profiles into diagnostic techniques to improve the clinical use of precision restorative therapy. We investigate the progression from crude fecal microbiota transplantation (FMT) to next-generation synthetic microbial consortia (SynComs), MEVs, and molecular postbiotics, providing a mechanism-based approach to controlling multi-organ comorbidities in companion animals.

## 2. The Host–Microbe Collaboration: Physiology of the BA Pool

The vertebrate BA pool represents a dynamic, circulating signal network maintained by the combined activity of two distinct genomes: the host’s hepatic pathways and the gut microbiome’s enzymatic refinery. In companion animals, this functional unit—conceptualized as the collaborative metabolome [14,15]—operates through a high-efficiency enterohepatic circulation (EHC) that recycles the BA pool approximately 4 to 12 times daily in healthy subjects [56]. The efficiency of this process ensures that while the total BA pool is relatively small, the daily flux through the intestine is substantial, providing continuous signaling input to both mucosal and systemic receptors. Understanding the healthy physiology of this collaboration is a precondition for distinguishing adaptive fluctuations from the pathological signatures of metabolic failure [15,17,57].

### 2.1. Hepatic Synthesis and the “Taurine Imperative”

BA synthesis initiates in the hepatocytes, where cholesterol undergoes catabolism via two primary pathways. The “classic” (neutral) pathway, initiated by cholesterol 7α-hydroxylase (CYP7A1), typically accounts for the majority of synthesis, while the “alternative” (acidic) pathway involving sterol 27-hydroxylase (CYP27A1) provides a secondary route. In canines, CA serves as the dominant primary species (as they are predominantly 12α-hydroxylators), occurring at significantly higher concentrations than CDCA—a physiological ratio that becomes progressively skewed toward CDCA during chronic hepatobiliary dysfunction or cholestasis [18,58]. This higher CA:CDCA ratio renders the canine BA pool naturally more hydrophilic and less cytotoxic than that of humans, a factor that must be considered when translating hepatotoxicity data between species [1,17]. In feline medicine, the serum profile is uniquely sensitive to nutritional status. Acquired taurine depletion leads to a marked increase in unconjugated BAs in serum, reflecting a failure of hepatic conjugation pathway that precedes clinical signs of deficiency [59]. The stability of this structural profile across mammalian taxa points to deep evolutionary conservation as a primary determinant, independent of direct dietary patterns [60,61].

Understanding the primary pool requires looking at its species-specific composition. Dogs and cats are obligate taurine conjugators [17,62,63]. Unlike humans or omnivorous rodents, which possess the enzymatic flexibility to utilize glycine when taurine availability is restricted, they lack significant bile acid-CoA:amino acid N-acyltransferase affinity for glycine. Simultaneous profiling across multiple biological matrices confirms that while unconjugated species dominate the fecal pool (95–99%), healthy canine serum and plasma are characterized by a predominance of taurine-conjugated BAs (approx. 68–75%), establishing these blood-based matrices as the primary readout for assessing host hepatic conjugation efficiency [17,18,64]. Comprehensive surveys across the *Ursidae* and related carnivores confirm that biliary BAs are conjugated solely with taurine via N-acyl linkage, regardless of specific dietary niches [61,65]. This “taurine imperative” renders the BA pool uniquely sensitive to dietary AA composition and microbial deconjugation activity. In the healthy state, over 98% of biliary BAs are taurine-conjugated (T-CA, T-CDCA). This specific modification lowers the pKa of the molecules to approximately 1.5, ensuring that they remain fully ionized as bile salts in the acidic environment of the proximal duodenum. This ionization prevents premature passive absorption, maintaining high intraluminal concentrations for lipid emulsification until the molecules reach the active ASBTs of the distal ileum [66,67,68].

### 2.2. Postprandial (PP) Dynamics: The Biphasic Kinetic Response and Motility Drivers

Following cholecystokinin (CCK)-mediated release into the duodenum, the BA pool moves in a kinetic pattern determined by gallbladder contractility and intestinal transit time (TT). In healthy dogs, PP serum BA concentrations follow a characteristic biphasic curve, as opposed to a single peak [69,70]. Targeted LC-MS/MS profiling shows that specific species, such as taurocholic acid (TCA), act as reliable markers of this flux, directly reflecting the neurohormonal gallbladder ejection [64,71,72,73].

The first phase, occurring 1 to 2 h after feeding, is driven by dietary fat-induced CCK release [74]. This neurohormonal contraction triggers emptying in a volume-dependent manner, though rapid emptying requires reaching specific dietary fat thresholds [75,76]. This response is highly sensitive to the site of nutrient delivery. Psáder et al. (2012) found that while intragastric delivery evokes maximal contraction (<30%), intrajejunal administration results in a significantly blunted response (~10%), confirming that proximal duodenal stimulus is necessary for the full surge. Such physiological variance is important for interpreting PP BA peaks in clinical settings, especially in patients receiving tube feeding [77].

Chronic dietary patterns also modulate this sensitivity. For instance, dogs fed high-fat, high-cholesterol diets show reduced gallbladder motility and decreased CCK sensitivity, alongside a shift toward hydrophobic BAs like taurochenodeoxycholic acid (TCDCA) [78].

The second phase, typically seen 3 to 8 h post ingestion, represents active BA reabsorption from the terminal ileum and its recycling to the liver [18,79]. This delayed peak relies on intestinal motility—specifically the interdigestive migrating motor complex—and ileal ASBT density [80,81,82]. Dyssynchronous or blunted peaks can therefore serve as early indicators of intestinal dysmotility or ileal malabsorption, often appearing before overt clinical signs such as hypoalbuminemia or chronic diarrhea [18,69,83].

Functionally, contractile capacity can act as a diagnostic discriminator. Ultrasound monitoring of cholagogue-induced emptying (e.g., via a fatty meal) reliably differentiates non-obstructive icterus (>40% contraction) from complete biliary obstruction (<5% contraction), offering a non-invasive alternative to more complex diagnostics [76]. While these dynamics were traditionally established through serum analysis and volumetry, the use of direct intraluminal techniques—such as the endoscopically guided nasojejunal chyme withdrawal described by Pápa et al. (2009)—offered a practical means to observe these processes directly within the intestinal lumen [84].

### 2.3. The Microbial Reactor: Deconjugation and Functional Conversion

**Figure 1:**
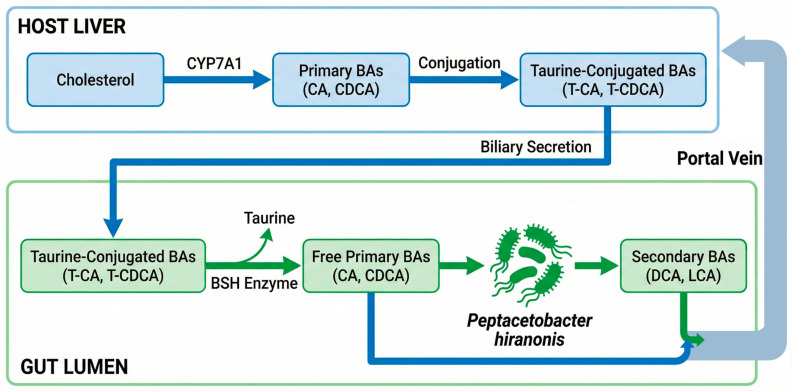
The collaborative host-microbial bile acid metabolism in companion animals. The liver synthesizes primary bile acids (CA, CDCA) from cholesterol, which are obligately conjugated with taurine before secretion. In the distal intestine, gut bacteria first deconjugate them via BSH. Subsequently, a specialized guild, dominated by *Peptacetobacter hiranonis* carrying the *bai* operon, mediates 7α-dehydroxylation, converting primary BAs into critical anti-inflammatory secondary BAs (DCA, LCA), which are then recirculated. Abbreviations: CYP7A1, 7α-hydroxylase; BA, bile acid; *bai*, bile acid-inducible operon; BSH, bile salt hydrolase; CA, cholic acid; CDCA, chenodeoxycholic acid; DCA, deoxycholic acid; LCA, lithocholic acid.

The process is finalized in the distal gut, where the fraction of BAs that escapes ileal reabsorption is subjected to extensive microbial modification. This “metabolic refinery” involves two distinct enzymatic tiers (Figure 1) [16,35].

#### 2.3.1. Gateway Deconjugation: The Role of Bile Salt Hydrolase (BSH) Enzyme

The first stage involves deconjugation, which is performed by the BSH enzyme. This process separates the taurine moiety from the steroid core, resulting in free (unconjugated) BAs. BSH activity is seen in many bacterial phyla, including *Lactobacillus*, *Bifidobacterium*, *Enterococcus*, and *Bacteroides*. It is important to note that the keystone species *Peptacetobacter hiranonis* also possesses the BSH gene, linking deconjugation and 7α-dehydroxylation capacities within the same guild [16,85,86]. While deconjugation is a physiological prerequisite for subsequent modifications, the premature or excessive activity of BSH-active bacteria—as seen in microbiota-related modulation-responsive enteropathy (MrMRE, characterized by dysbiosis (often historically termed small intestinal bacterial overgrowth, SIBO, or antibiotic-responsive enteropathy, ARE, though these entities likely represent distinct or overlapping pathologies rather than synonyms)—can lead to premature deconjugation in the proximal gut. The resulting unconjugated BAs possess inferior emulsifying properties and cause direct mucosal irritation, leading to fat malabsorption and chronic diarrhea [18,87,88,89,90]. The clinical relevance of BSH activity makes it a potential therapeutic target in metabolic liver disease. Modulating this enzymatic step provides a mechanism to regulate lipid metabolism and alleviate steatohepatitis by shifting the splanchnic flux of hydrophobic BAs [85].

#### 2.3.2. 7α-Dehydroxylation and the Bai Operon Guild

The most biologically impactful modification is 7α-dehydroxylation, a multi-step enzymatic process that converts PBAs into SBAs. CA is transformed into DCA, while CDCA becomes LCA. Unlike BSH activity, this specific metabolic capacity is restricted to a highly specialized and fragile guild of bacteria harboring the *bai* operon [20,35,91]. The transition from PBAs to SBAs represents a major signaling shift, as SBAs possess significantly higher affinity for the TGR5 and FXRs, which modulate host inflammation and metabolism. The microbial composition responsible for this conversion varies between species. While the human 7α-dehydroxylation community is diverse—including *Lachnoclostridium* (formerly *Clostridium*) *scindens*, *Lachnoclostridium* (formerly *Clostridium*) *hylemonae*, and *Paraclostridium* (formerly *Clostridium*) *sordellii* [20,92]—the canine and feline ecosystems rely on a more specialized structure. Although *C. scindens* is occasionally detected, *P. hiranonis* fulfills the primary metabolic function in dogs and cats [21,23,91].

In parallel with this primary pathway, specific bacteria such as *Ruminococcus* spp. mediate reversible 7α/β-epimerization, converting primary CDCA into the tertiary bile acid UDCA and its taurine conjugate (TUDCA). While less abundant in the healthy canine pool than in ursids or humans, these hydrophilic metabolites serve as cytoprotective modulators that counterbalance the cytotoxicity of hydrophobic SBAs [16,93].

### 2.4. The Keystone Species: P. hiranonis

This bacterium dominates the 7α-dehydroxylation guild in dogs and cats [91]. As an obligate anaerobic bacterium, *P. hiranonis* is exceptionally sensitive to oxygen exposure, which can rapidly diminish vegetative cell viability. This necessitates specialized handling of clinical samples, including rapid, air-tight storage and chilled transport, to ensure accurate detection via molecular methods [22,24]. While this bacterium acts as the collaborative anchor of the entire gut–liver axis, it is notably unstable during storage. Correa Lopes et al. (2025) found rapid viability loss in frozen samples without cryoprotectants like glycerol. Hence, DNA-based detection in historical banked samples or FMT preparations may not reflect 7α-dehydroxylation capacity [24]. Empirical evidence confirms a strict threshold-dependency for this species: when *P. hiranonis* abundance falls below 10^5^ gene copies per gram of feces—typically following broad-spectrum antibiotic treatment (e.g., metronidazole) or severe inflammatory insults—the biotransformation process is disrupted. While 10^5^ is the lower reference limit, functional loss of conversion typically occurs below 10^4^ [91,94]. This leads to the metabolic extinction of SBAs and a concurrent accumulation of unconverted PBAs—a state defined as the core signature of microbial collapse [19,25,87].

Recent characterizations of the microbiome’s response to the BA pool have demonstrated that dominant bacterial phyla exhibit distinct growth and metabolic responses to specific BA species, further highlighting the sensitivity of the “microbial refinery” to changes in pool composition [95].

### 2.5. Microbially Conjugated Bile Acids (MCBAs): The “Fifth Mechanism” and Host Interaction

Beyond the classic primary and secondary categories, the collaborative metabolome generates a diverse array of metabolites with complex signaling roles. For decades, BA conjugation was viewed solely as a hepatic process utilizing glycine or taurine. Recent discoveries have shown that microbially conjugated bile acids (MCBAs)—representing a process of reconjugation by the gut microbiota—have redrawn the map of the collaborative metabolome [96,97]. This phenomenon is now recognized as the “fifth mechanism” of BA modification, distinct from deconjugation, oxidation, epimerization, and 7α-dehydroxylation [96].

#### 2.5.1. The Transamidation Pathway and Biosynthesis

MCBA synthesis occurs via a specific transamidation mechanism catalyzed by the bacterial BSH enzyme. Previously known only as a deconjugating enzyme, recent structural analyses confirmed that BSH also possesses N-acyltransferase activity. Compelling evidence suggests these metabolites are synthesized via this transamidation activity, effectively transforming a traditionally destructive enzyme into a signaling generator. This allows the microbiota to couple the steroid backbone of CA or CDCA with non-canonical AAs such as phenylalanine, tyrosine, histidine, leucine, isoleucine, glutamine or tryptophan [98,99]. Key producing species include *Enterocloster* (formerly *Clostridium*) *bolteae*. The resulting molecules—e.g., phenylalanocholic acid (Phe-CA), tyrosocholic acid (Tyr-CA), and leucocholic acid (Leu-CA)—significantly expand the chemical and functional diversity of the BAP [96,98,99].

#### 2.5.2. Functional Significance and Systemic Signaling

These MCBAs are not metabolic waste products but specific, high-affinity ligands that function as potent systemic messengers or remote sensors of gut status. Unlike host-produced taurine conjugates, MCBAs possess unique receptor affinities that allow the microbiome to re-program the host’s metabolic signaling. In this context, tertiary BAs, such as UDCA and its taurine conjugate, tauroursodeoxycholic acid (TUDCA), act as cytoprotective agents; unlike hydrophobic SBAs, UDCA stabilizes mitochondrial membranes and prevents apoptosis, acting as a molecular chaperone to mitigate cellular stress [100,101,102].

The presence of such metabolites in systemic circulation suggests that the microbiome actively communicates its functional state to the entire organism. For instance, Phe-CA and Tyr-CA activate FXR and TGR5, influencing hepatic lipid metabolism and systemic inflammation from a distance [103,104]. Hence, in states of collaborative collapse, the loss of this conjugation diversity likely contributes to the multi-system metabolic failure observed in chronic diseases [15,96].

Shifts in MCBA levels may serve as predictive biomarkers. Research in pediatric Crohn’s disease suggests clinical relevance for veterinary “therapy-resistant” enteropathies [105]. Recent studies have also linked the trajectory of early-life MCBA levels to the risk of islet autoimmunity [97]. This raises the possibility that early microbiome programming in puppies and kittens could determine long-term resilience against autoimmune and metabolic diseases, highlighting the developmental importance of this fifth mechanism of conjugation [97].

#### 2.5.3. Physiological Variation: The Host Size Factor

The efficiency of microbial conversion is fundamentally influenced by the host’s physical and physiological parameters. Deschamps et al. (2024) utilized the canine mucosal artificial colon (CANIM-ARCOL) in vitro model to demonstrate that large-breed dogs naturally exhibit a more efficient microbial conversion profile (characterized by a lower primary-to-secondary (P/S) ratio) than small-breed dogs. This is primarily attributed to the longer intestinal TTs inherent to larger breeds, which favor extensive 7α-dehydroxylation by the resident microbiota. Large-breed models also show significantly higher production of short-chain fatty acids (SCFAs) and gas, indicating a more mature fermentative profile. This finding emphasizes that diagnostic thresholds for microbial collapse must be interpreted in the context of breed-specific physiological baselines to avoid misinterpreting natural variation as pathological failure [106].

To synthesize the diverse metabolic roles discussed throughout this chapter, Table 1 provides a functional classification of the collaborative metabolome and outlines the clinical relevance of its key components.

## 3. Molecular Pathophysiology: BA Receptors as the Interface of the Collaborative Axis

The biological identity of the BA pool is fundamentally defined by its ability to function as a high-affinity systemic signaling network. This communication is mediated by a diverse array of host receptors that operate as metabolic and immunological sensors, continuously translating the chemical flux of the collaborative metabolome into coordinated physiological responses. The most relevant signaling hubs involve the nuclear FXR and the membrane-bound TGR5. The disruption of these pathways—whether through the metabolic extinction of SBA ligands or the toxic accumulation of PBAs—represents the molecular basis of the collaborative collapse and its associated multi-organ pathologies [8,9,10].

It is crucial to acknowledge species-specific variations in this signaling architecture. Unlike humans, where the FXRβ isoform is a pseudogene, dogs express an FXRβ receptor, suggesting a broader transcriptional regulatory capacity. TGR5 expression in canines has been documented in the pancreas, implying a direct role for BAs in modulating pancreatic secretion and insulin sensitivity beyond the classical incretin effect [15].

### 3.1. FXR: The Homeostatic Brake and Barrier Guardian

FXR is a metabolic nuclear receptor expressed at high levels in the hepatocytes and the enterocytes of the distal small intestine [3,107]. It functions as the primary intracellular sensor for BA levels, maintaining the equilibrium of the enterohepatic circulation through complex transcriptional feedback loops that prevent metabolic overload.

#### 3.1.1. The Gut–Liver Feedback and Synthesis Control

In the ileum, the activation of FXR by PBAs (specifically CDCA and CA) triggers the expression and secretion of fibroblast growth factor 19 (FGF19), the canine ortholog of rodent FGF15 [108]. Once released into the portal circulation, FGF19 binds to the FGFR4/β-Klotho complex on hepatocytes, initiating a phosphorylation cascade that suppresses the transcription of CYP7A1—the rate-limiting enzyme in BA synthesis. This synthesis brake is further bolstered by the induction of the small heterodimer partner, which represses the transactivation of BA biosynthetic genes. This is a crucial homeostatic mechanism. In states of hepatobiliary spillover or type 2 BAD, this feedback loop is frequently impaired or bypassed due to ileal malabsorption or inflammation. The resulting unregulated synthesis leads to a sustained flood of PBAs into the colon, which overwhelms the local microbial conversion capacity and causes direct mucosal damage [31,109,110].

#### 3.1.2. Mechanical and Antimicrobial Barrier Protection: The Mucosal Shield

Beyond its role in synthesis control, intestinal FXR acts as a barrier guardian with a broad range of protective effects. Its activation promotes the transcription of genes encoding indispensable tight junction proteins, including occludin, zonula occludens-1, and various claudins, which collectively maintain the mechanical integrity of the intestinal wall against bacterial translocation [8,10,111]. FXR also directly influences the biochemical environment of the mucus layer by modulating mucin secretion and inducing the expression of antimicrobial peptides such as angiogenin-1 and cathelicidins. These peptides provide a chemical first line of defense against pathobiont overgrowth. FXR activation suppresses the pro-inflammatory nuclear factor kappa-light-chain-enhancer of activated B cells (NF-κB) signaling pathway and prevents the assembly of the NOD-, LRR-, and pyrin domain-containing protein 3 (NLRP3) inflammasome within the intestinal epithelium, effectively blunting the local response to microbial triggers [8,111,112]. The loss of this FXR-mediated protection, a hallmark of the collaborative collapse, directly facilitates the “leaky gut” phenotype and the systemic low-grade inflammation observed in canine CE and PLE [26,27,113].

### 3.2. TGR5 and the Gut–Immune Axis: The Molecular Architecture of Signaling Starvation

TGR5 is a membrane-bound receptor coupled to Gs proteins, characterized by a broad tissue distribution that includes intestinal L-cells, resident macrophages (e.g., Kupffer cells), and the central nervous system [5,114]. Unlike FXR, TGR5 exhibits its highest affinity for SBAs, specifically LCA and DCA, effectively positioning it as a direct molecular sensor of microbial metabolic health [115,116].

#### 3.2.1. The Incretin Response and Glucose Homeostasis

Activation of TGR5 on the basolateral membrane of enteroendocrine L-cells triggers an intracellular increase in cyclic adenosine monophosphate (cAMP) and subsequent protein kinase A (PKA) signaling. This cascade promotes the synthesis and secretion of GLP-1, a multi-system incretin hormone that enhances insulin secretion, slows gastric emptying, and regulates satiety [9,117]. In patients suffering from microbial collapse, where SBA concentrations fall below the operational threshold, this incretin response is severely blunted. The resulting glycemic dysregulation, insulin resistance, and altered appetite are frequently observed in companion animals with chronic GI diseases or EPI [9,57]. This axis is of particular importance in feline medicine; as obligate carnivores with a glucose metabolism prone to insulin resistance, cats may specifically benefit from TGR5-mediated GLP-1 stimulation as a therapeutic strategy for feline diabetes and obesity-associated metabolic dysfunction [118]. This PP axis also influences hepatic immune tolerance. The synchronized release of peptide hormones and BAs coordinates nutrient disposal while maintaining a tolerogenic environment to prevent inflammatory responses against dietary antigens [119].

#### 3.2.2. Macrophage Polarization and Inflammasome Regulation

A major function of TGR5 in the context of the collaborative collapse is its role as a master regulator of innate immunity, driving a shift that extends beyond simple cytokine suppression. In resident macrophages and dendritic cells, TGR5 activation initiates a signaling shift that suppresses the release of pro-inflammatory cytokines such as tumor necrosis factor α (TNF-α), interleukin (IL)-1β, and IL-6. This immunomodulatory effect is mechanically distinct: under physiological conditions, the binding of SBAs to TGR5 triggers cAMP elevation and PKA activation. PKA phosphorylates the cAMP-response element-binding protein (CREB), which subsequently inhibits the transcriptional activity of NF-κB [9,120]. Recent research has identified the direct regulation of the NLRP3 inflammasome by this pathway. TGR5 activation promotes the PKA-mediated ubiquitination and subsequent degradation of NLRP3, preventing its assembly and the maturation of IL-1β and IL-18 [10,49,111,120,121].

**Figure 2:**
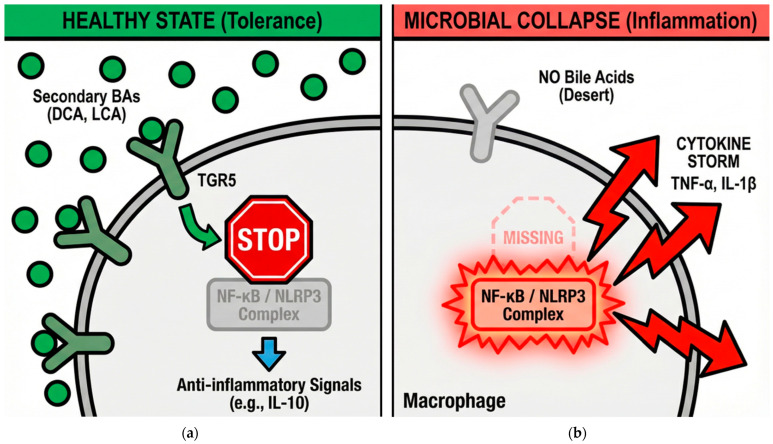
The molecular architecture of signaling starvation in macrophages. (**a**) Healthy state: Sufficient secondary bile acids (green dots) activate the membrane-bound TGR5 receptor. This induces intracellular cAMP-PKA signaling, which acts as a molecular brake on the pro-inflammatory NF-κB and NLRP3 inflammasome complexes, maintaining immune tolerance; (**b**) Microbial collapse: The loss of *P. hiranonis* creates a “bile acid desert”. Without ligand activation, the TGR5 receptor fails to inhibit the inflammatory cascades, leading to the activation of NF-κB/NLRP3 and a subsequent “cytokine storm” (TNF-α release), which drives chronic inflammation. Abbreviations: DCA, deoxycholic acid; LCA, lithocholic acid; cAMP, cyclic adenosine monophosphate; NF-κB, nuclear factor kappa B; NLRP3, NLR family pyrin domain containing 3; PKA, protein kinase A; TGR5, transmembrane G protein-coupled receptor 5; TNF-α, tumor necrosis factor alpha; IL, interleukin.

Consequently, TGR5 signaling promotes a functional repolarization from the pro-inflammatory M1 macrophage phenotype to the anti-inflammatory, pro-resolving M2 phenotype. This tonic, SBA-mediated signaling acts as a continuous anti-inflammatory brake preserving mucosal tolerance and preventing the systemic propagation of inflammatory signals from the gut. When this brake is lost due to the eradication of *P. hiranonis* and the subsequent loss of SBAs, the innate immune system enters a state of hyper-responsiveness. Microbial products like lipopolysaccharides can then initiate unchecked inflammatory cascades, driving both localized mucosal damage and the systemic decline observed in cardiovascular and renal “gut–X” pathologies [8,37,91]. This mechanism explains why bacterial conversion failure is both a marker of gut disease and a systemic pro-inflammatory engine. Figure 2 illustrates the contrast between homeostatic signaling and the inflammatory cascade following ligand depletion.

#### 3.2.3. Neutrophil Extracellular Traps (NETs) and Barrier Defense

Beyond macrophage regulation, the collaborative metabolome directly influences neutrophil biology, a key driver of tissue damage in acute inflammation. Recent data have demonstrated that LCA specifically inhibits the formation of NETs via the NLRP3-GSDMD (gasdermin D) signaling axis [49,122,123]. This indicates that the loss of LCA-producing bacteria, such as *Odoribacter splanchnicus* and *P. hiranonis*, removes a crucial molecular brake on neutrophil-mediated tissue injury, further compromising the intestinal barrier during active colitis [10,124].

#### 3.2.4. Adaptive Immunity: Fine-Tuning the Regulatory T Cells (Treg)/T Helper 17 (Th17) Balance

BAs function as gatekeepers of immune homeostasis, regulating the crosstalk between innate and adaptive responses. Specific BA receptors on immune cells modulate cytokine release and differentiation, linking metabolic signaling to immune networks within the gut–liver axis [10]. This regulatory role is particularly evident in the adaptive immune response, where BAs fine-tune the delicate balance between Tregs and inflammatory Th17 cells. Targeted metabolomics have identified specific SBA derivatives, including 3-oxo-LCA and isoallo-LCA, as modulators of T-cell differentiation (3-oxo-LCA inhibits Th17 differentiation by binding to the transcription factor RORγt, while isoallo-LCA promotes Treg differentiation by stimulating the expression of forkhead box P3 (FoxP3), a key transcription factor, via a mitochondrial reactive oxygen species (mitoROS)-dependent mechanism) [104,125,126,127].

In canine chronic enteropathy (CE), the significant depletion of these specific iso- and oxo-bile acids correlates with disease severity, suggesting a failure of this adaptive tolerance mechanism [125,128,129]. Recent studies on semi-synthetic nor-UDCA have suggested that it inhibits Th17 cells while promoting their conversion into Treg phenotypes, a mechanism that provides a therapeutic strategy for autoimmune bowel diseases [49,130].

#### 3.2.5. Innate Lymphoid Cells (ILCs) and Antiviral Competence

The immune interface extends to viral defense mechanisms. Recent research has highlighted the connection between BAs and ILCs, specifically ILC3s, which rely on microbial metabolites to regulate IL-22 and IL-26 production for epithelial homeostasis [123]. Moreover, specific BAs exhibit direct antiviral properties. In a porcine deltacoronavirus infection model, LCA was shown to effectively inhibit viral replication independent of classical receptor (FXR, TGR5) signaling, potentially by modifying membrane integrity or viral entry mechanisms [49,123,131,132]. This implies that an optimal secondary BAP provides a layer of mucosal protection against both bacterial pathobionts and specific enteric viruses.

### 3.3. MCBA Signaling: The Mas-Related G Protein-Coupled Receptor Member E (MRGPRE) and Systemic Metabolic Control

The signaling repertoire of the collaborative metabolome extends to novel G-protein-coupled receptors beyond TGR5. Recent evidence identified tryptophan-cholic acid (Trp-CA) as a potent agonist for the orphan MRGPRE [133]. The binding of Trp-CA to MRGPRE stimulates (GLP-1) secretion from intestinal L-cells via Gs-cAMP and β-arrestin-1 pathways [133].

This mechanism carries substantial clinical implications for companion animals. By enhancing GLP-1 secretion, Trp-CA improves glucose tolerance and insulin sensitivity, addressing the metabolic dysregulation often observed in obese or diabetic patients. Trp-CA and its synthetic analogs thereby represent potential therapeutic targets for metabolic syndrome and diabetes mellitus (DM) in dogs and cats, where GLP-1 deficiency is a common pathophysiological factor [9,133]. This highlights that the fifth mechanism of conjugation is a key component of the host’s incretin response system.

### 3.4. Pathophysiology of the “Two Faces” of Collapse

The molecular failure of the BA axis manifests through two distinct, often synergistic mechanisms that define the clinical progression of companion animal diseases.

#### 3.4.1. Ligand Starvation: The Signaling Shortfall

The microbial collapse profile is fundamentally defined by a state of ligand deficiency. The eradication of the 7α-dehydroxylation guild results in a systemic and luminal absence of secondary metabolites. This creates a state of “TGR5/FXR signaling starvation” where the host is deprived of the inputs required to maintain mucosal immune quiescence, barrier stability, and metabolic signaling [19,26]. This signaling deficit is not simply a consequence of disease but a primary engine of chronicity, facilitating the progression of intestinal inflammation and its systemic extensions, such as the gut–heart axis in MMVD [37].

#### 3.4.2. Cytotoxic Overload: PBA Detergency and Pore Formation

In contrast, the hepatobiliary spillover profile involves the toxic accumulation of PBAs (particularly conjugates of CA and CDCA) in the colon. At high intraluminal concentrations, PBAs exert direct detergent effects on the colonic epithelium. These hydrophobic molecules induce pore formation and cause the rearrangement of tight junction proteins such as occludin, significantly increasing mucosal permeability and allowing the influx of luminal toxins [134,135]. PBAs act as potent secretagogues via the activation of membrane receptors and the cystic fibrosis transmembrane conductance regulator (CFTR) channel, inducing the severe, watery secretory diarrhea characteristic of BAD [33,110,136]. Extracellular vesicles secreted by cholangiocytes and hepatocytes regulate the local inflammatory response and ductal proliferation within the biliary tree; these signals can initiate cholangiopathies prior to the systemic spillover event [137].

## 4. Measuring the Collapse: A Functional Diagnostic Assessment

Diagnosing BA dysmetabolism must move from viewing BAs as static liver markers to recognizing them as dynamic endocrine signals. Traditional tests rely on serum total bile acid (TBA), but this approach does not capture the collaborative collapse. A multi-tiered system using fecal matrices is required. To accurately assess this complex system, a hierarchical diagnostic approach is recommended: serum markers provide an overview of hepatic clearance and synthesis (top-down view), while fecal profiling characterizes the functional capacity of the microbiome (bottom-up view).

### 4.1. Serum Profiling: Systemic Signaling and Limitations

Serum TBA remains the gold standard for assessing hepatic extraction efficiency and identifying PSS or primary hepatocyte failure [83,138,139]. However, interpretation is complicated by breed-specific baseline elevations. Kim et al. (2023) confirmed that clinically healthy Maltese dogs regularly exhibit pre- and postprandial SBA concentrations exceeding established reference intervals without evidence of macroscopic shunting or hepatocellular injury. This suggests a breed-specific vascular leakiness or altered extraction efficiency that necessitates careful contextualization of results in small-breed screening [140]. Similarly, while the SBA stimulation test achieves nearly 100% sensitivity for detecting PSS, its specificity for identifying surgical resolution is significantly lower (19–44%) [29].

Modern serum LC-MS/MS profiling allows for the simultaneous quantification of 15–20 individual BA species, providing a high-resolution view of the systemic signaling pool [64]. Latest data have identified specific shifts within this pool—specifically significant elevations in conjugated PBAs, such as TCA and glycocholic acid (GCA)—as early-warning indicators of progressive hepatic fibrosis and hepatocellular injury [31,141]. These metabolic changes often precede manifest clinical signs like hyperbilirubinemia. However, because over 95% of the BA pool is reabsorbed in the distal ileum before reaching the microbial “refinery” of the colon, serum levels provide limited information regarding the functional status of the intestinal microbiome [18,87,142,143,144]. Monma et al. (2022) empirically confirmed this limitation in dogs with CE. Their data show while fecal BA profiles showed severe dysbiosis and SBA collapse, serum concentrations often remained within reference intervals, thus not reflecting the intraluminal metabolic failure [145].

### 4.2. Fecal BA Profiling: Defining the Intraluminal Fingerprint

While serum TBA represents the net efficiency of hepatic extraction, fecal profiling provides an immediate readout of the intraluminal metabolic refinery [17,146]. Fecal BA profiling serves as the direct metabolic fingerprint of the collaborative axis and remains the only clinically viable method to quantify the transition from primary to secondary BAs [147]. In a healthy state, fecal BAs are characterized by a pronounced dominance (>90%) of secondary metabolites like DCA and LCA. The shift towards a primary-dominant profile—even in the presence of normal liver function—is the definitive biochemical signature of microbial collapse [25,87,144]. The transition to precision diagnostics requires high-resolution analytical platforms. Liquid chromatography–tandem mass spectrometry (LC-MS/MS) is the current standard. The diagnostic power of LC-MS/MS is significantly enhanced by electrospray ionization, a technique particularly well suited for this application as it enables the sensitive detection of the diverse biotransformations executed by intestinal bacteria [61,147]. Unlike traditional enzymatic assays, LC-MS/MS allows for the simultaneous quantification of primary, secondary, and tertiary BAs with high specificity, ensuring that subtle shifts in the P/S ratio are captured with diagnostic accuracy [73,147].

### 4.3. The Dysbiosis Index (DI) and the P. hiranonis Benchmark

The development of the canine DI represents a landmark advancement in veterinary diagnostics [128,148,149]. While *Turicibacter* is crucial to the commensal-host lipid network [150], *P. hiranonis* provides the index’s principal functional measure. Research has established a clear threshold-dependent relationship between the abundance of this species and the metabolic capacity of the gut. When *P. hiranonis* levels drop below the reference interval of 10^5^, the 7α-dehydroxylation capacity is compromised. Functional studies indicate that the complete loss of 7α-dehydroxylation capacity typically initiates at abundances below 10^4^ gene copies/g, leading to a precipitous decline in SBA production [19,26,91]. Because the DI targets bacterial genomic DNA instead of viable microorganisms, the assay remains valid even when cells are no longer viable, provided genomic integrity is preserved. Chilled or frozen transport is therefore required to prevent nuclease-mediated DNA degradation—independent of bacterial viability—ensuring accurate results from archived or transported samples [149,151,152].

#### Diagnostic Precision: qPCR vs. Sequencing

It is important to note that 16S rRNA gene sequencing often underreports or misses *P. hiranonis* due to primer limitations and relative abundance issues. Therefore, targeted qPCR is the preferred method for identifying microbial collapse. This provides a window for intervention—such as FMT or targeted dietary shifts—before systemic comorbidities arise [57,129,151,153]. It is noteworthy that in many cases, the correlation between *P. hiranonis* abundance and clinical scores tends to normalize as clinical signs improve.

### 4.4. The P/S Ratio

The most definitive measurement of the collaborative collapse is the P/S ratio. In healthy dogs and cats, the P/S ratio is highly skewed towards secondary BAs (typically < 0.1). In patients with CE or EPI, this ratio undergoes a dramatic inversion [19,25]. The accumulation of PBAs coupled with the loss of DCA and LCA creates the “two faces” of pathology: TGR5/FXR signaling derangement and cytotoxic detergent effects.

### 4.5. Targeted Metabolomics and Signaling Hubs: FXR and TGR5 Readouts

The ultimate goal of diagnostics is to assess the state of receptor signaling. Modern diagnostic panels integrate receptor activity readouts to better characterize the overall state of this signaling axis.

TGR5 ligand availability: By measuring the fecal SBA pool (LCA and DCA), clinicians can use this as an indirect proxy for the status of the TGR5-GLP-1 incretin axis. The loss of these specific ligands—essential for glucose homeostasis and anti-inflammatory braking—marks a transition from localized GI disease to a systemic pro-inflammatory state [116,117].

FXR signaling and FGF15/19: While direct measurement of ileal FXR activity is not yet clinically available in veterinary patients, fecal BA profiling provides an important clue. An excess of fecal PBAs often reflects a failure of the FXR-FGF15/19 feedback loop, suggesting unregulated synthesis and hepatobiliary spillover [108,109,112].

### 4.6. Diagnosing the Systemic Extension: The “Gut–X” Biomarkers

Diagnostic profiling is now expanding to capture the “gut–X” axes, where specific systemic metabolite shifts serve as early-warning indicators for distal organ injury and multi-system metabolic failure.

Gut–heart markers (LCA/UDCA Ratio & TMAO): Canine myxomatous mitral valve disease (MMVD) involves dysbiosis characterized by more than just diversity loss. Li et al. (2021) reported a specific disruption in CDCA transformation, resulting in a suppressed lithocholic acid (LCA) to ursodeoxycholic acid (UDCA) ratio. This shift correlates with ACVIM stage progression and left atrial enlargement. Additionally, elevated Trimethylamine N-oxide (TMAO) levels—driven by microbial diet processing—are linked to myocardial fibrosis and adverse remodeling, suggesting these metabolites serve as prognostic markers for cardiac progression [37,154].

Gut–Kidney Markers: Feline CKD is characterized by a shift toward proteolytic fermentation. Yaqub et al. (2025) linked the depletion of glycolytic taxa, such as *Peptococcaceae*, to the systemic accumulation of gut-derived uremic toxins, notably indoxyl sulfate (IS) and p-cresol sulfate. These metabolites—which increase alongside SBA deficiency—accelerate tubulointerstitial fibrosis through direct nephrotoxicity, driving a maladaptive renal-gut loop [40,155].

Gut–brain markers: Metabolic profiling provides a non-invasive means to assess neuro-metabolic status in epileptic patients in dogs with drug-resistant idiopathic epilepsy, a distinct fecal profile has been identified, characterized by altered BA ratios and increased inflammatory markers—specifically involving the loss of anti-inflammatory *Blautia* and *P. hiranonis*. Assessing these neuro-inflammatory thresholds allows clinicians to identify a collapsed “neuro-metabolic shield”. Monitoring fecal SBA ratios thus identifies systemic metabolic contributions to the lowering of the seizure threshold, offering a molecular basis for managing drug-refractory cases [53]. Systemic BAPs further bridge the gap between gut dysbiosis and neurological status. The serum P/S ratio serves as a clinical window into the neuro-metabolic shield where a decline in circulating SBAs is associated with cognitive impairment and depression-like phenotypes driven by gut–liver axis failure [156].

### 4.7. Diagnostic Pitfalls and Breed-Specific Considerations

Interpretation of these functional markers requires careful consideration of host physiological variation. As demonstrated by Deschamps et al. (2024), large-breed dogs naturally exhibit a more mature fermentative profile and higher conversion efficiency (lower P/S ratios) than small-breed dogs. This suggests that a P/S ratio that might be considered borderline in a small terrier could be pathological in a large breed like a German Shepherd [106,157]. Clinicians must be aware of the “washout effect” in severe diarrhea, where rapid TT may prevent even a healthy microbiome from completing the conversion process, leading to a transiently primary-dominant profile that does not necessarily reflect a true collaborative collapse.

### 4.8. Future Diagnostic and Research Frontiers: Isotope Tracing

As we move toward precision metabolomics, future canine and feline research should prioritize the mapping of MCBAs across disease states. Applying ex vivo 13C-isotope tracing (e.g., incubating feces with 13C-labeled amino acids) will be essential to confirm these transamidation pathways in veterinary patients [99,133,158,159,160]. If validated, specific MCBA profiles could serve as early-warning biomarkers for metabolic syndrome, intestinal barrier failure, or the onset of collaborative collapse, potentially allowing for postbiotic supplementation using synthetic MCBA analogs to normalize host signaling without complex bacterial engraftment [23,99,154,155,161].

Beyond metabolites, circulating MEVs represent a diagnostic frontier in gut–brain axis monitoring. These vesicles cross the blood–brain barrier to deliver specific molecular cargo, providing non-invasive biomarkers for neuroinflammation and systemic signaling dysfunction [55].

## 5. Clinical Manifestations I: The Microbial Collapse Profile (Gut–Centric)

The systemic failure of the host–microbiome collaborative metabolome, described physiologically in Chapter 2 and molecularly in Chapter 3, manifests clinically as a distinct and measurable biochemical signature. This signature, defined by a collapse in SBA production, serves as a common pathogenic factor across a spectrum of primary GI diseases in companion animals. This microbial collapse profile is consistently observed in CE, its severe PLE, and EPI. The transition from a healthy, SBA-dominant environment to a PBA-rich signaling problem is not just a consequence of intestinal inflammation but a primary engine of chronicity [25,26].

### 5.1. CE: The Mechanics of TGR5 Signaling Dysregulation

CE in dogs—encompassing food-responsive, antibiotic-responsive, and steroid-responsive inflammatory bowel disease (IBD) variants—represents the classic clinical manifestation of the microbial collapse [162,163]. While metabolic failure is a core feature, recent data have indicated a gradient of underlying changes; some dogs exhibit severe BA dysmetabolism and dysbiosis, while others maintain a more stable profile, suggesting distinct pathophysiological subtypes within the CE spectrum [129]. A substantial body of evidence confirms that these dogs exhibit a significant and consistent inversion of the fecal P/S ratio, marking the transition to dysbiosis [25,164].

Recent targeted metabolomics have quantified the extent of this collapse: while healthy dogs maintain an SBA pool constituting approximately 88–96% of total unconjugated BAs, this proportion plummets to 28–64% in dogs with CE and an elevated DI [129]. Comito et al. (2023) further demonstrated that these dogs exhibit severe impairments in their metabolic BAP, specifically characterized by a significant decrease in DCA and a concurrent increase in CA and CDCA, regardless of the clinical sub-type. This biochemical signature quantifies the collapse of the ecosystem and the resulting signaling insufficiency [129,164].

#### 5.1.1. The Core Signature and Receptor Starvation: The Immunological Cascade

In CE, the eradication of the *bai* operon-carrying active guild, primarily *P. hiranonis*, leads to the systemic and luminal extinction of DCA and LCA [19,143]. This metabolic shift induces a state of TGR5 receptor starvation. As established in Chapter 3, TGR5 activation provides a tonic anti-inflammatory brake on the mucosal immune system. Without these high-affinity SBA ligands, the resident immune landscape undergo a pathological shift: resident macrophages lose their ability to maintain the tolerogenic M2 phenotype and instead polarize towards a pro-inflammatory M1 state. This facilitates the unchecked release of pro-inflammatory cytokines such as TNF-α, IL-1β, and IL-6, creating a localized cytokine storm that further degrades barrier function and promotes epithelial apoptosis [8,117,120].

This signaling gap creates a maladaptive, self-perpetuating cycle: the lack of SBA-mediated NF-κB inhibition promotes chronic mucosal inflammation, which further alters the luminal environment—increasing oxidative stress and oxygen tension—making it increasingly hostile to the strictly anaerobic *P. hiranonis*. Porcine intestinal epithelial cell models confirm this pathway, as oxidative stress directly impairs tight junction integrity—notably through claudin-4 disruption—and induces the secretion of pro-inflammatory cytokines such as IL-6 and IL-8 [165,166,167,168,169]. The conversion failure thereby drives the disease progression, explaining why many CE patients fail to achieve long-term remission with anti-inflammatory therapy alone unless the microbiome’s metabolic capacity is restored [57,116,153].

#### 5.1.2. Expanded Metabolomic Insight: Iso- and Oxo-Bile Acids

The novelty of recent research lies in the discovery that this signaling discrepancy is far broader than previously understood. Blake et al. (2025) utilized a specialized 30-bile acid panel to demonstrate that CE dogs also exhibit a significant depletion of oxo-BAs (e.g., 3-oxo-LCA) and iso-BAs, which normally constitute up to 30% of the healthy fecal BA pool. These oxidized and epimerized metabolites are increasingly recognized as specific modulators of mucosal T-cell responses and ligands for nuclear receptors. Their loss suggests a multi-layered failure of the metabolic shield required to maintain oral tolerance and prevent the autoimmune-like triggers characteristic of IBD [125,129,148].

#### 5.1.3. The Lipid-Sterol-Bile Acid Triangle: The “Multifactorial Pathogenic Loop”

In protein-losing enteropathy (PLE), BA dysmetabolism correlates directly with systemic failures in lipid and sterol homeostasis. Targeted metabolomics in dogs with inflammatory PLE show a fecal profile defined by SBA depletion and a deficit in phytosterols (e.g., sitostanol), occurring concurrently with a marked accumulation of cholesterol and long-chain fatty acids (LCFAs) [26]. This “lipid-BA triangle” suggests a complex interaction wherein the loss of SBA signaling impairs intestinal lipid processing and chylomicron formation. The resulting luminal retention of undigested fats acts as a substrate for dysbiotic fermentation, promoting the growth of lipolytic pathobionts that further compromise the mechanical barrier. Additionally, the disruption of the enterohepatic circulation leads to the loss of trophic signals required for lacteal integrity, exacerbating lymphangiectasia. The degree of SBA depletion in PLE correlates strongly with hypoalbuminemia and clinical severity scores (CIBDAI), positioning the P/S ratio as a superior prognostic marker compared to traditional inflammatory indices [26,113,170].

### 5.2. Pre-Clinical Dysmetabolism: The “Smoking Gun” in At-Risk Breeds

A key question in CE pathogenesis is whether BA dysmetabolism is a cause or a consequence of inflammation. Evidence for the former comes from the study of soft-coated Wheaten Terriers (SCWTs), a breed genetically predisposed to PLE and protein-losing nephropathy (PLN) [171].

A landmark longitudinal study by Tolbert et al. (2025) investigated asymptomatic SCWTs and identified a subclinical state of dysmetabolism. These dogs exhibited significant increases in intestinal permeability (measured via higher lactulose:galactose AUC) and alterations in BA metabolism before the onset of clinical diarrhea or hypoalbuminemia. Specifically, 25% of asymptomatic SCWTs harbored a collapsed *P. hiranonis* population and an abnormal fecal P/S ratio (defined as <50% total SBAs), mirroring the lipid dysmetabolism (e.g., lower sitostanol) seen in dogs with clinical PLE [172]. This finding provides the “smoking gun”. It suggests that the microbial collapse is a pre-clinical event that predisposes the gut to inflammatory triggers. This identifies a crucial window for early intervention and preventative screening in at-risk breeds.

### 5.3. Exocrine Pancreatic Insufficiency (EPI): The pH-Driven Collapse

In EPI, the microbial collapse follows a distinct path fueled by the absence of pancreatic enzymes and the resulting shift in intraluminal chemistry. The lack of bicarbonate secretion in EPI leads to a significant decrease in proximal small intestinal pH. This acidic environment inhibits the enzymatic activity of the *bai* operon apparatus, specifically the 7α-dehydroxylase enzyme, even if *P. hiranonis* is still taxonomically present [144,173].

Massive amounts of undigested proteins and carbohydrates creates a nutrient-rich substrate for the overgrowth of acid-tolerant, fermentative bacteria (e.g., specific *Lactobacillus* strains or *Bifidobacterium*), which can outcompete the specialized BA converters [25,173]. EPI dogs present with a “double hit” of maldigestion: they lack host lipase activity and simultaneously lose the microbe-driven SBA signaling required for mucosal immune quiescence [15]. This explains why many EPI patients suffer from persistent dysbiosis and secondary intestinal inflammation even after receiving adequate pancreatic enzyme replacement therapy [87,173].

### 5.4. The Feline Parallel: Conserved Pathophysiology and Antibiotic Fragility

The microbial collapse is a conserved mechanism across small animal carnivores [15]. Recent data confirmed that cats with CE (subtyped as IBD or small cell lymphoma) exhibit patterns consistent with the pathological signature as their canine counterparts: high DI scores, severe depletion of *P. hiranonis*, and a near-total collapse of SBA production [149,174,175]. Giordano et al. (2024) further corroborated this, who noted significantly altered LCFA and unconjugated BAPs in feline CE cohorts [176].

The fragility of this system in cats is particularly evident during medical intervention. Martini et al. (2025) showed that metronidazole treatment in healthy cats causes a precipitous drop in *P. hiranonis* and SBA production (*p* < 0.0001) that persists for weeks after treatment stops. This antibiotic-induced collapse is accompanied by an increase in fecal lactate and serum uremic toxins (e.g., IS), reinforcing the link between dysbiosis and systemic metabolic failure [176,177,178]. These findings reinforce the clinical mandate to avoid empirical antibiotic therapy, as it often exacerbates the metabolic derangement it seeks to treat, potentially accelerating systemic comorbidities.

## 6. Clinical Manifestations II: The Hepatobiliary Spillover and Systemic Axes

The disruption of BA metabolism extends far beyond the GI tract, representing a multi-organ endocrine failure. While the microbial collapse profile (Chapter 5) defines primary enteropathies by a deficiency in metabolic signaling, a distinct pathology occurs when host-driven hepatobiliary dysfunction or dysbiotic metabolites reach the systemic circulation. This hepatobiliary spillover represents a containment failure where host dysfunction floods the gut with PBAs, acting as a central driver for extra-intestinal pathologies. This dysregulation establishes a “gut–X” disease concept that links the intestine to the kidneys, heart, and central nervous system [37,40,141,179].

### 6.1. Hepatobiliary Spillover and Bile Acid Diarrhea (BAD)

A specific form of BA dysmetabolism occurs in dogs with chronic liver disease, particularly those affected by biliary tract disease (BTD) or acute cholestatic insults [30,31]. Functional ultrasonography with cholagogue testing facilitates the confirmation of physical patency in icteric patients, as complete obstruction eliminates the contractile response typical of hepatocellular disease [76]. Unlike the SBA depletion seen in CE, this profile is marked by the fecal accumulation of PBAs, such as CA and CDCA, which overwhelms the reabsorptive capacity of the ileum and the conversion capacity of the distal gut. Translational evidence from human and rodent models of steatotic liver disease further supports this, identifying conjugated PBAs like TCA as consistent markers across fibrosis stages (F0–F4), correlating directly with hepatocellular injury (ALT enzyme elevation) and stellate cell activation [141].

#### 6.1.1. Mechanism: Host-Driven Pathogenesis and Secretory Toxicity

In this profile, the microbial refinery often remains structurally intact, with *P. hiranonis* concentrations frequently reaching healthy thresholds [30]. The failure is physiological: impaired biliary flow regulation or transporter saturation (ASBT) forces an unregulated spillover of PBAs into the colon. These molecules act as potent secretagogues; they trigger Cl^−^ and water secretion via the CFTR channel and induce high-amplitude colonic contractions that accelerate transit, manifesting clinically as severe secretory diarrhea [33,109,113].

#### 6.1.2. Diagnostic Breakthroughs: 7α-Hydroxy-4-cholesten-3-one C4 and FGF19

Diagnosing BAD has always posed a barrier in veterinary medicine owing to the absence of precise indicators. Contemporary methods enable the distinction between biliary spillover and microbial collapse, with serum C4—a persistent intermediary of bile acid synthesis—acting as a dependable biomarker. Increased serum C4 levels signify a compensatory rise in hepatic production due to malabsorption or excessive loss [180,181]. Validation studies associate elevated C4 levels with fecal BA loss, providing a non-invasive substitute for complex fecal examinations.

In conjunction with C4, FGF19 serves as a functional readout of ileal feedback. In humans, low FGF19 signals Type 2 (idiopathic) BAD. While canine-specific assays are evolving, measuring the FGF19/C4 ratio helps clinicians differentiate between primary synthesis defects and secondary malabsorption [110]. The ^75^Se-labeled tauroselcholic acid (SeHCAT) test —a radioactive retention assay—is rarely available in veterinary settings; functional fecal profiling (comprising total quantity and the P/S ratio) combined with serum C4 provides a practical metabolic fingerprint for identifying this pathology [181,182,183].

#### 6.1.3. Clinical Validation and the Efficacy of Sequestration

The clinical relevance of this mechanism was confirmed by Habermaass et al. (2024), noting a 50% diarrhea prevalence in BTD-affected dogs compared to only 16% in dogs with other liver diseases. Further validation is provided by the efficacy of pharmacological sequestration. Toresson et al. (2025) demonstrated in a retrospective case series that a subset of enteropathy dogs—refractory to standard anti-inflammatory protocols but exhibiting high fecal PBAs—achieved sustained clinical remission over a follow-up of 5–47 months through the use of BA sequestrants (e.g., cholestyramine). In human medicine, Barbara et al. (2025) showed that while cholestyramine is effective, its powder formulation frequently causes poor palatability and low compliance in dogs and cats. Colesevelam, a second-generation agent, provides a higher affinity binding profile and a better safety profile with fewer GI side effects. Its availability in tablet form makes it the preferred clinical choice for long-term management of BAD [30,94,110,184]. This confirms that spillover is a distinct metabolic entity requiring metabolic intervention rather than immune modulation [30,110,183,185].

### 6.2. Portosystemic Vascular Anomalies: The Systemic Bypass

PSSs represent a definitive “containment failure” where the hepatic extraction is physically bypassed. Congenital PSS—classified as extrahepatic PSS in small/toy breeds and intrahepatic PSS in large breeds—diverts blood from the splanchnic circulation directly into the systemic venous system [28]. This bypass results in altered signaling; the systemic circulation is flooded with high concentrations of primary conjugated BAs (e.g., TCA, TCDCA) and gut-derived toxins like ammonia that should remain confined to the enterohepatic circuit [28,29].

A clinical distinction must be made between macroscopic shunts and microscopic hepatic portal circulation abnormalities, such as primary hypoplasia of the portal vein (formerly termed microvascular dysplasia). Schermerhorn et al. (1996) confirmed that while both conditions result in elevated serum BAs due to shunting at different levels, MVD-affected dogs (notably Cairn Terriers) are often asymptomatic and lack the characteristic hematologic abnormalities (microcytosis) and ammonium urate crystalluria seen in congenital PSS [28,186]. The metabolic fingerprint of PSS extends beyond the fecal P/S ratio. It reflects a failure of hepatic clearance that allows the collaborative metabolome to trigger the astrocyte–microglia-mediated neuroinflammatory activation characteristic of HE [54,187].

### 6.3. Gallbladder Mucocele (GBM): A Systemic Metabolic Failure

GBM is increasingly recognized not as an isolated anatomical defect, but as a systemic metabolic failure linked to the gut–liver feedback loop. Dogs with GBM possess a distinct fecal BA pattern defined by excessive GCA and a deficit in T-β-muricholic acid (T-β-MCA). This metabolic shift is tightly coupled with a specific dysbiosis, primarily an overgrowth of *Enterobacteriaceae* (e.g., *E. coli*). The loss of specific metabolic signals likely impairs ileal FXR signaling, which then fails to regulate biliary fluidity and mucin secretion, leading to the formation of the viscous, pro-inflammatory bile characteristic of mucoceles [188,189,190,191,192]. Evidence for a localized biliary microbiome has redefined this context; Lee et al. (2026) found that bile duct dysbiosis—marked by an enrichment of *Fusobacterium* and *Enterococcus*—differentiates malignant from benign biliary diseases, pointing to a local microbial driver of cholangiopathies [193]. Dietary patterns appear to exacerbate this condition. Kakimoto et al. (2017) demonstrated that high-cholesterol diets increase biliary TCDCA concentrations while concurrently impairing CCK-mediated gallbladder emptying, thereby promoting the stasis necessary for mucocele formation [78].

The specific loss of T-β-MCA is particularly significant, as this metabolite functions as a potent naturally occurring FXR antagonist in the ileum; its depletion may deregulate the synthesis brake, leading to an unregulated influx of BPAs that further destabilizes the biliary environment [189,194].

BAs provide definitive evidence of anatomical gallbladder failure. Pascual Moreno et al. (2025) established that the total BA concentration ratio between abdominal fluid and serum is the most accurate diagnostic metric for identifying bile peritonitis. A fluid and serum BA ratio >2 is 100% sensitive and highly specific for gallbladder rupture, significantly outperforming traditional bilirubin-based ratios, which often yield false negatives in early or partial rupture cases [195,196].

In felines, BA dysmetabolism is central to the pathophysiology of neutrophilic cholangitis, even though GBM is less frequent than in dogs. In this condition, the reflux of duodenal contents and bacteria triggers the inflammatory cascade, with the subsequent biliary dysbiosis perpetuating the inflammatory state. These findings identify the restoration of the gut–liver axis as a priority for long-term management [15].

### 6.4. The Gut–Kidney Axis: Uremic Cross-Talk and Renal Fibrosis

In feline CKD, gut-derived metabolites actively modulate renal survival through a bidirectional feedback loop. Rowe et al. (2024) showed that cats in International Renal Interest Society (IRIS) Stages 2–4 exhibit a collapse of the SBA pool and the loss of *P. hiranonis*. This results in a systemic deficiency of UDCA and its taurine conjugate [40].

In the healthy state, UDCA provides a fundamental molecular brake on renal injury by inhibiting mitochondrial-mediated apoptosis and reducing endoplasmic reticulum stress in tubular cells. Its extinction leaves the renal parenchyma vulnerable to accelerated fibrogenesis. Clinically, serum reductions in UDCA and CDCA correlate with declining glomerular filtration rates in advanced kidney disease, independent of other comorbidities [197]. The retention of uremic toxins (e.g., IS) simultaneously alters the intestinal environment, exerting a direct inhibitory effect on the *bai* operon-carrying guild, further suppressing SBA production. This creates a maladaptive cycle, renal failure drives dysbiosis, and the resulting BA dysmetabolism accelerates renal fibrosis, ensuring that neither organ can spontaneously recover without targeted intervention. This axis appears to involve the lower urinary tract as well. Recent profiling in cats with idiopathic cystitis identified distinct microbiome alterations, indicating that gut-derived metabolic stress can drive inflammatory pathologies throughout the urogenital system [34,41,178,198].

### 6.5. The Gut–Heart Axis: Hemodynamic Drivers and TMAO in MMVD

The link between cardiac performance and gut metabolism follows a pathological cycle prominently observed in canine MMVD. As cardiac output falls, chronic intestinal hypoperfusion and mucosal edema—termed congestive enteropathy—trigger a progressive increase in the DI [34,37].

This environment precipitates the depletion of *P. hiranonis* and the proliferation of choline-fermenting bacteria. The resulting systemic rise in TMAO correlates with myocardial fibrosis and adverse cardiac remodeling in dogs [37]. Clinical data in advanced MMVD cases confirm that increased gastrointestinal permeability allows for bacterial translocation, driving systemic inflammation—evidenced by elevated C-reactive protein (CRP)—and establishing the gut as a primary source of systemic toxicity [199]. Also, the loss of TGR5-activating SBAs facilitates a chronic inflammatory state that accelerates valvular degeneration. Under physiological conditions, these SBA ligands suppress pro-inflammatory cytokine release from macrophages by inhibiting the NF-κB pathway—a systemic immunomodulatory mechanism needed for cardiometabolic protection that is lost during microbial collapse [9,23,37,49,200,201,202,203].

### 6.6. The Gut–Brain Axis: Neuroinflammation, Epilepsy, and Behavioral Phenotypes

BA dysmetabolism is no longer confined to GI signs but is increasingly linked to neurological and behavioral outcomes. BAs function as neuroactive steroids crossing the BBB to modulate neuroinflammation and synaptic plasticity. In dogs with drug-resistant idiopathic epilepsy, a collapsed fecal SBA pool and a loss of anti-inflammatory *Blautia* and *P. hiranonis* have been definitively identified [53,204]. The enteric nervous system mediates this crosstalk by relaying microbial signals—including SCFA concentrations—via vagal afferent pathways to modulate neuroinflammation [205].

BA signaling modulates stress response and cognitive plasticity through a specific TGR5-mediated pathway. Evidence from recent mechanistic studies indicates that SBA-mediated activation of TGR5 receptors on neurons and microglia upregulates BDNF via the CREB signaling cascade [206]. Accordingly, the physiological conversion failure—characterized by the loss of SBAs—leads directly to a downregulation of BDNF. This molecular deficit offers a plausible explanation for the anxiety, cognitive decline, and “depression-like” behaviors frequently observed in companion animals with CEs, establishing an inherent neuro-metabolic link between the gut and the limbic system [206,207,208,209,210,211,212].

Beyond structural pathologies, the axis influences complex behavioral phenotypes. Sacchettino et al. (2025) evidenced that dogs with generalized fear exhibit significant alterations in serum metabolites associated with gamma-aminobutyric acid (GABA) and glutamate neurotransmission, alongside a distinct shift in BA metabolism [213,214]. These metabolic shifts are particularly relevant to canine anxiety and aggression, where the gut microbiome modulates hypothalamic–pituitary–adrenal axis responsiveness [215,216]. This suggests that the loss of SBA-mediated neuroprotection lowers the threshold for stress-related disorders. Furthermore, gut dysbiosis—specifically *Lactobacillus* and *Catenibacterium* alterations—is linked to aggression and phobic behaviors, potentially mediated by the modulation of serotonergic pathways [207,211,213,217,218].

In severe states such as HE, the synergy between high circulating ammonia and altered BAPs triggers a cytokine storm driven by a pathobiological astro-glial interaction. In this process, pro-inflammatory BAs sensitize microglia to ammonia-induced stress, leading to the exaggerated release of IL-6 and TNF-α. This crosstalk induces cytotoxic edema and facilitates neuronal apoptosis. In addition, the loss of neuroprotective BAs, such as TUDCA, which normally stabilize mitochondrial membranes, leaves the BBB vulnerable to oxidative insults, accelerating cognitive decline [51,54,187,209,219]. Microbiota-derived extracellular vesicles also mediate this axis by crossing the BBB to deliver neuroprotective or pro-inflammatory signals directly to glial cells, particularly during traumatic or metabolic brain injury [220]. Using multi-omic profiling, Mottawea et al. (2025) identified these vesicles as specialized carriers that shield neurotransmitters—including GABA, glutamate, and dopamine—during transport across host barriers [221].

### 6.7. Metabolic Extensions: Metabolic Dysfunction-Associated Steatotic Liver Disease (MASLD), Diabetes, and Physiological Variation

The gut–liver axis finds its most complex expression in the pathophysiology of metabolic syndromes, where intestinal dysbiosis acts as a primary instigator of systemic dysfunction. This relationship is best characterized in MASLD and insulin-dependent DM. Current models describe a failure of the “tripartite barrier”—comprising mechanical, immune, and microbial defenses—that exposes the liver to a constant influx of gut-derived insults. Loss of barrier integrity facilitates the translocation of pathogen-associated molecular patterns (PAMPs), triggering chronic hepatic inflammation and fibrosis [10,222].

This collaborative collapse of the gut–liver alliance is evident in canine diabetes. Jergens et al. (2019) demonstrated that dogs with naturally occurring, insulin-dependent DM exhibit a distinct BA dysmetabolism, characterized by an increased percentage of primary CA and a significant decrease in secondary lithocholic acid LCA compared to healthy controls [223,224].

Beyond BA perturbations, dysregulated metal metabolism is a hallmark of the steatotic liver. Recent cohort studies have identified hepatic copper accumulation as a potent predictor of fibrosis progression. Copper overload appears to induce mitochondrial dysfunction via oxidative stress, driving the transition from simple steatosis to severe pathology [225].

#### 6.7.1. The Host Size Factor: TT and Conversion Efficiency

Interpreting these metabolic shifts requires rigorous accounting for the host’s physiological baseline, particularly regarding canine body size. Deschamps et al. (2024), utilizing the CANIM-ARCOL in vitro model, established that large-breed dogs naturally exhibit more efficient microbial conversion (lower P/S BA ratio) than small breeds. This efficiency is driven by the longer intestinal TTs inherent to larger breeds, which favor extensive 7α-dehydroxylation [106].

Although TBA concentrations are often lower in large-breed models, their production of SCFA and gas is significantly higher, indicating a shift toward a more mature fermentative profile. Thus, diagnostic thresholds for dysmetabolism must be breed-contextualized, applying uniform reference ranges risks misinterpreting these physiological variations as pathological failure [106,157,226,227].

#### 6.7.2. Integrated Metabolomic Profiling and Diagnostic Strategy

Diagnosing the progression of metabolic liver disease is complicated by the heterogeneous nature of the organ itself. Experimental models of steatosis reveal significant inter-lobar variability in gelatinase activity and oxidative stress, with the left lobe often exhibiting metabolic profiles distinct from the right [228]. This heterogeneity suggests that single-site liver biopsies may underestimate disease severity, emphasizing the critical need for serum-based metabolic surrogates.

To address this, an integrated diagnostic strategy is proposed. As Rey et al. (2025) showed, combining BA profiling with markers of fatty acid and AA catabolism provides a comprehensive systemic fingerprint. A concurrent reduction in SCFAs and alterations in tryptophan-derived metabolites allows for the detection of metabolic fragility before gross clinical organ failure occurs [170].

For clinical decision support, we propose a multi-modal approach combining TBA pairs, targeted fecal BA profiling, and the DI. This triangulation allows clinicians to distinguish between primary gastrointestinal and primary hepatobiliary pathologies, compensating for the limitations of focal biopsies [18,94,170,224].

### 6.8. The Gut–Skin Axis: Microbial Modulation of Atopy

The influence of the collaborative metabolome extends to the cutaneous barrier. Considering the prevalence of canine AD in Central Europe [229], research has increasingly focused on the metabolic drivers behind the disease. Dysbiosis—specifically the depletion of butyrate-producing taxa like *Fusobacterium*—correlates with the severity of AD in dogs. The gut–skin axis operates through immunological crosstalk. Microbial metabolites regulate the differentiation of Tregs which, in turn, mitigate systemic pruritus and allergic inflammation. Restoring this axis through FMT or targeted metabolic support offers a novel adjunctive strategy for managing refractory dermatologic cases [45,46,47,230,231].

Recent clinical data have validated these mechanisms. Oral FMT trials in atopic dogs have shown a 92% clinical response rate by day 56, with a significant reduction in both pruritic intensity and scores on the Canine AD Extent and Severity Index, 4th iteration (CADESI-04). This therapeutic success is rooted in the restoration of microbial diversity—specifically the recovery of butyrate-producing taxa that are typically depleted in chronic dermatopathologies. These findings mirror human clinical advancements where FMT suppresses systemic inflammation by shifting the gut environment toward an anti-inflammatory state. By promoting the expansion of butyrate-producers and enhancing the differentiation of FoxP3+ Tregs, the transplant triggers a surge in IL-10 production, which directly dampens the cutaneous allergic cascade [231,232,233,234,235,236,237].

**Figure 3:**
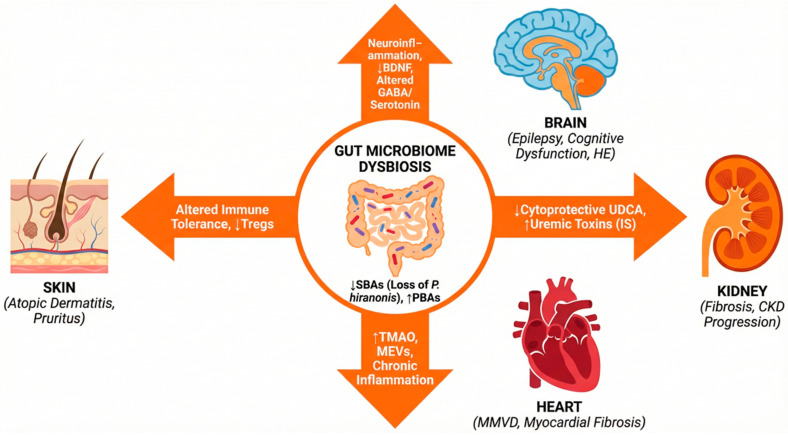
The Gut–X network: systemic consequences of bile acid dysmetabolism. A disrupted intestinal collaborative metabolome—characterized by the loss of *P. hiranonis* and secondary bile acids—acts as a central driver of pathology in distant organs. Through the translocation of pro-inflammatory metabolites (e.g., TMAO, uremic toxins), MEVs, and the loss of protective signaling (e.g., UDCA or TGR5 ligands), the gut influences the progression of cardiac, renal, neurological, and cutaneous diseases. This metabolic crosstalk extends to skeletal, respiratory, and reproductive systems, reflecting the microbiome’s systemic reach. The upward (↑) and downward (↓) arrows indicate an increase or decrease in the respective parameters. Abbreviations: BDNF, brain-derived neurotrophic factor; CKD, chronic kidney disease; MMVD, myocardial fibrosis; GABA, gamma-aminobutyric acid; HE, hepatic encephalopathy; IS, indoxyl sulfate; MEVs, microbial extracellular vesicles; PBA, primary bile acid; SBA, secondary bile acid; TGR5, transmembrane G protein-coupled receptor 5; TMAO, trimethylamine N-oxide; Tregs, regulatory T cells; UDCA, ursodeoxycholic acid.

Beyond simple engraftment, this “metabolic recalibration” suggests that the gut–skin axis can be therapeutically modulated to resolve refractory skin inflammation where conventional therapies fail. These diverse systemic manifestations reflect the broad impact of BA dysmetabolism across distal organ systems, as illustrated in the “Gut–X” network summarized in Figure 3. Table 2 details the associations between microbial metabolites, pathological mechanisms, and their clinical consequences across these organ systems.

## 7. Therapeutic Modulation: Restoring the Collaborative Metabolome

The recognition of BA dysmetabolism as a primary pathogenic driver changes clinical intervention in companion animals. Evidence establishes two distinct pathological profiles requiring stratified therapy. First, microbial collapse (seen in CE, PLE, and MMVD), defined by a failure of SBA production, and second, hepatobiliary spillover (seen in BTD and BAD), defined by an excess of colonic PBAs. This mechanistic distinction is fundamental for clinical success, as strategies effective for restoring SBA function are often contraindicated for hepatobiliary spillover due to the osmotic and secretory toxicity of excess PBAs [4,109,110,148,149,184].

### 7.1. Iatrogenic and Dietary Modulators: The Origins of Failure and Recovery

Before launching therapy, clinicians must identify and alleviate factors that exacerbate BA dysmetabolism, primarily iatrogenic antibiotic usage and pro-inflammatory dietary patterns. Antibiotics commonly used in veterinary gastroenterology, such as metronidazole and tylosin, are potent modulators of the collaborative metabolome. Their administration triggers the rapid eradication of the 7α-dehydroxylating guild, specifically *P. hiranonis* [57,238]. This iatrogenic collapse results in a “bile acid desert”—a profile dominated by PBAs with a near-total absence of anti-inflammatory SBAs. Németh et al. (2024) and Martini et al. (2025) have demonstrated that this metabolic depletion can persist for months after drug cessation, accompanied by the accumulation of uremic toxins and fecal lactate, directly inducing clinical signs and complicating the diagnosis of the underlying primary disease [87,178].

Diet serves as the primary long-term modulator. The impact of dietary structure is pronounced; research comparing raw meat-based diets (BARF) to traditional extruded commercial diets has revealed distinct microbial and metabolomic signatures. However, data remain inconsistent across studies, and observing minor diet-induced fluctuations does not necessarily imply pathology. Furthermore, interpretations of raw diet studies are often confounded by variable fat content and pathogen loads, warranting caution. Dogs fed raw diets typically exhibit higher microbiome richness [239] and altered fecal BAPs, characterized by increased levels of unconjugated PBAs compared to those on high-carbohydrate extruded pet food [239,240,241]. Yang et al. (2025) recently analyzed how macronutrient balance—mainly the protein-to-carbohydrate ratio—and functional ingredients like fiber and probiotics influence microbial diversity and metabolism [242]. Within this context, the specific protein source appears to be a key factor. For instance, healthy dogs fed insect protein (black soldier fly larvae) showed favorable microbiome shifts and enhanced SCFA production compared to standard diets [243]. While Mioto et al. (2025) noted varied SCFA responses depending on inclusion rates, the consensus suggests that novel proteins may better support a stable microbiome. Overall, these studies indicate that gut health depends as much on chemical complexity and nutrient ratios as it does on the diet’s physical form [242,243,244,245].

Recent translational insights from a pig model have demonstrated that even a moderate increase in dietary fat (12% weight/weight) can induce regional microbiota alterations and metabolomic remodeling along the digestive tract prior to any systemic metabolic changes or obesity development. This finding supports the “smoking gun” theory in companion animals, suggesting that dietary fat modulation acts as an early trigger for BA dysmetabolism before clinical symptoms appear [246]. These observations are consistent with the data of Kakimoto et al. (2017), showing that lipid overload specifically modifies hepatic gene expression (e.g., CYP7A1) and drives the BA pool toward a more hydrophobic composition. Pilot data, however, indicate that targeted caloric restriction can reverse these shifts in the serum BA pool, demonstrating the inherent plasticity of the host-microbial metabolic axis in response to energy intake [78,247].

Mason et al. (2025) revealed that a “Western-style” diet (high-fat, high-carbohydrate, low-fiber) induces low-grade systemic inflammation (elevated high sensitivity CRP, NF-κB) and a significant increase in fecal CA in only 30 days [248]. Conversely, dietary fiber remains a significant management tool, though its effects are highly substrate-specific. Salavati Schmitz et al. (2024) demonstrated that psyllium supplementation, unlike resistant starch, maintains microbiome richness in healthy dogs, supporting its role as a stable substrate [249]. Psyllium acts as a dual-action tool: its prebiotic properties support the *bai* guild and increase SCFA production [87,250,251,252], while its mechanical sequestration triggers a compensatory hepatic synthesis overshoot that activates protective FXR signaling [251,253,254]. However, fiber alone cannot normalize SBA production if the keystone converter, *P. hiranonis*, has been entirely eradicated, highlighting the need for combined microbial and substrate intervention [57,118,249].

### 7.2. Strategy I: FMT and Microbial Restoration

FMT represents the most direct method to re-establish the 7α-dehydroxylating pathway. In veterinary medicine, FMT has evolved from an experimental procedure to a cornerstone of “mechanism-based” therapy. The objective is no longer simple “repopulation” but the re-establishment of metabolic guilds—specifically *P. hiranonis* carrying the *bai* operon—to restart bile acid transformation and signaling.

#### 7.2.1. Standardizing Protocols: Preparation and Stability

Despite its potential, donor material viability remains a frequently overlooked limiting factor. Recent studies confirmed that while lyophilized (freeze-dried) oral capsules offer efficacy comparable to fresh suspensions and superior logistical feasibility, the processing method is paramount [24,255,256,257]. Due to the susceptibility of *P. hiranonis* to oxidative stress and freeze–thaw damage, FMT preparations stored in standard saline at −80 °C lose most viable cells within weeks. Maintaining metabolic efficacy requires the use of specific cryoprotectants, such as 10% glycerol or trehalose, which preserve the structural integrity of this obligate anaerobe for up to 6 months. While viability of strict anaerobes decreases during lyophilization compared to glycerol-preserved freezing, recent data suggest that metabolic efficacy may be preserved depending on storage duration and the severity of the recipient’s disease. Ensuring the survival of these organisms allows FMT to re-seed the gut with a viable community capable of *bai* operon expression, effectively bypassing the “signaling starvation” of the host [24].

Clinical outcomes reflect this precision. In a large-scale trial involving 171 dogs with CE, a 30-day course of oral lyophilized FMT resulted in an 82% clinical response rate, effectively restoring SCFA production and microbial diversity [257].

Further studies are needed to fully validate the long-term clinical efficacy of oral capsules compared to endoscopic delivery. Supporting these findings, Vecchiato et al. (2025) confirmed that in CE dogs refractory to diet, FMT induces significant improvements in CIBDAI scores and SBA production, with 50% of responders remaining clinically stable for up to one year post procedure. In working dogs with chronic large bowel diarrhea, Alves et al. (2023) showed that FMT improves clinical signs, such as fecal consistency and CIBDAI, more effectively than high-dose psyllium (16 g/day) alone, although it is important to note that the control group treated with psyllium also exhibited clinical recovery, suggesting that supportive care alone can be effective in specific colitis phenotypes [257,258,259]. While Pérez-Accino et al. (2025) observed that a single rectal enema could provide symptomatic relief for approximately 10 weeks, the underlying microbial engraftment—specifically the stable colonization of *P. hiranonis*—often requires more intensive, repeated dosing protocols as advocated by Toresson et al. (2023) to achieve long-term metabolic normalization and facilitate steroid weaning [259,260,261].

#### 7.2.2. Dosing Strategy: The Move to Repeated FMT

**Figure 4:**
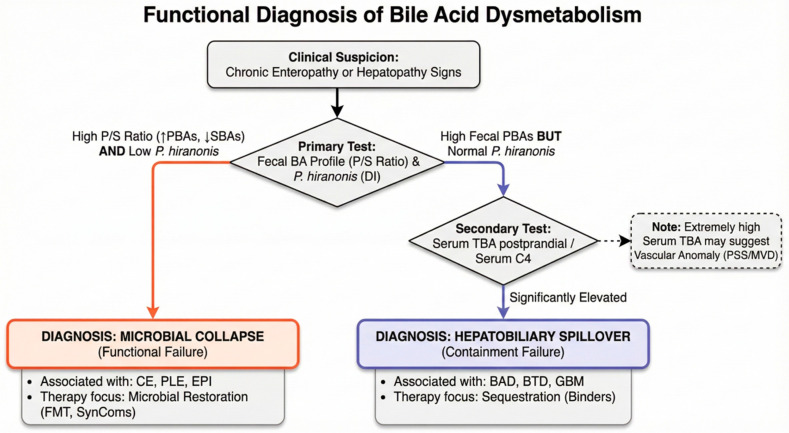
The temporal dynamics of metabolic collapse and restoration. Antibiotic administration (e.g., metronidazole) can induce the rapid eradication of the 7α-dehydroxylating guild, dropping *P. hiranonis* abundance below the effective threshold (<10^5^). This creates a “bile acid desert” dominated by primary bile acids (PBAs). Restoring of secondary bile acid (SBA) signaling often requires targeted intervention, such as fecal microbiota transplantation (FMT) or synthetic consortia (SynComs), to reestablish this functional guild and cross the metabolic threshold. Abbreviations: BAD, bile acid diarrhea; BTD, biliary tract disease; C4, 7α-hydroxy-4-cholesten-3-one; CE, chronic enteropathy; EPI, exocrine pancreatic insufficiency; FMT, fecal microbiota transplantation; GBM, gallbladder mucocele; MVD, microvascular dysplasia; PBA, primary bile acid; PLE, protein-losing enteropathy; P/S, primary-to-secondary bile acid ratio; PSS, portosystemic shunt; SBA, secondary bile acid; SynComs, synthetic microbial consortia; TBA, total bile acids.

Single-dose FMT often results only in transient engraftment, with the recipient’s microbiome reverting to dysbiosis within weeks. To achieve durable results, repeated FMT protocols have become the standard. Recent longitudinal data demonstrated that for dogs with refractory CE, repeated administration (e.g., weekly or bi-weekly induction followed by maintenance) significantly reduces the canine IBD activity index (CIBDAI, the standard clinical activity index for dogs with CE) and facilitates the tapering or discontinuation of corticosteroids [32]. For patients with structural defects or genetic susceptibility, intermittent “top-up” doses or continuous low-dose capsule supplementation may be required to prevent relapse [32,163]. The temporal dynamics of this iatrogenic collapse and the subsequent restoration of the *P. hiranonis* threshold are shown in Figure 4.

#### 7.2.3. Donor Screening and the DI

FMT success relies on donor quality. “Super-donors” are now selected based on metabolic capacity, not just pathogen absence. The canine and feline DI serves as the primary screening tool. Ideal donors must exhibit a normal DI (dogs < 0, cats < 1) and, crucially, a high *P. hiranonis abundance* (>10^5^ gene copies/g) to ensure the transfer of 7α-dehydroxylation capacity [128,255,262]. Some experts and evolving guidelines recommend rigorous screening for multidrug-resistant organisms, ensuring that the transplant does not inadvertently introduce resistance genes and pathogenes [255,263,264,265].

#### 7.2.4. Expanding Indications: “Gut–X” Applications

FMT applications now extend beyond chronic diarrhea. By modulating the gut–skin and gut–brain axes, restorative therapy is showing promise in extra-intestinal conditions. In AD, FMT can reduce pruritus and lesion severity, potentially by restoring butyrate-producing bacteria like *Fusobacterium* [45]. Similarly, in idiopathic epilepsy, normalizing the microbiome may reduce seizure frequency via the modulation of GABA and serotonin pathways [266,267]. Emerging research also highlights the oral–gut axis: at a mechanistic level, vesicles shed by oral pathobionts can translocate to the gut and enter systemic circulation, effectively spreading inflammation [268,269]. Jin et al. (2025) characterized this pathway, demonstrating that oral pathobionts (e.g., *Veillonella* spp.) expressing the *prtC* gene secrete a collagenase that degrades the intestinal barrier. This breach allows for their translocation to the liver, where they exacerbate fibrosis [270].

While less frequent in veterinary practice than in human medicine, FMT treats refractory *Clostridioides difficile* infections by restoring the SBA-producing guild to provide colonization resistance against the pathogen [24,257,265,271].

#### 7.2.5. Feline Specifics and Antibiotic Recovery

In cats, FMT significantly reduces uremic toxins and fecal lactate more effectively than dietary fiber alone following metronidazole treatment [178,272]. The success of these interventions is fundamentally tied to the viability of strict anaerobes within the transplant. Correa Lopes et al. (2025) highlighted that while lyophilization initially reduces viable cell counts, the remaining population of *P. hiranonis* remains stable over time, whereas glycerol-based freezing remains the gold standard for maintaining the high-density metabolic diversity required for functional restitution [24].

#### 7.2.6. Safety, Precision, and Limitations

While generally well-tolerated, Lee et al. (2025) reported mild to moderate adverse events in cats (e.g., transient vomiting, lethargy), underscoring the need for meticulous donor screening for both pathogens and a “healthy” metabolomic profile [273]. Furthermore, Marclay et al. (2022) cautioned that FMT might not accelerate recovery in all contexts, as recipient factors like existing inflammation and the provision of substrate (e.g., fiber) play major roles in engraftment success [274,275].

### 7.3. Strategy II: Managing PBA Excess (For BAD and Spillover)

This strategy applies to the hepatobiliary spillover profile, where the clinical goal is to mitigate the secretory and osmotic toxicity of excess colonic PBAs. The therapeutic standard for BAD is the use of bile acid sequestrants (BAS) such as cholestyramine or colesevelam. Toresson et al. (2025) validated this in a retrospective case series, showing significant clinical improvement sustained over a long-term follow-up of 5–47 months in enteropathy dogs with high fecal PBAs [94,276,277,278,279,280,281,282]. Because these agents bind various lipophilic compounds, administration should be separated from other oral medications by 2–4 h. Long-term therapy also necessitates monitoring for the malabsorption of fat-soluble vitamins (A, D, E, K) [277].

Complementary to pharmacological BAS, psyllium acts as a potent mechanical sequestrant. As a viscous hydrocolloid, psyllium traps luminal BAs, impeding their reabsorption in the terminal ileum. This interruption of the EHC triggers a distinct biphasic kinetic response, with PP serum BA peaks observed at ~2 h and 3–5 h [69,283,284]. Of note, in healthy dogs, this sequestration triggers a compensatory hepatic synthesis overshoot, which paradoxically increases total BA exposure while stabilizing fecal consistency. Furthermore, Bretin et al. (2023) established that psyllium-mediated shifts directly activate the FXR, providing protection against mucosal inflammation and colitis independent of prebiotic effects [253,254,285].

### 7.4. Strategy III: Pharmacological Bypass and the MCBA Horizon

Pharmacological bypass represents the next path in BA therapeutics, shifting the focus from microbial recovery to direct host receptor modulation.

#### 7.4.1. UDCA and TUDCA: The Cytoprotective Standard

UDCA and its taurine conjugate have long served as the standard of care for managing cholestasis and biliary sludge, primarily by promoting bile flow and stabilizing mitochondrial membranes [286,287,288]. TUDCA functions as a potent chemical cytoprotective agent and conformational stabilizer, capable of alleviating endoplasmic reticulum stress, stabilizing mitochondrial membranes to prevent the opening of the mitochondrial permeability transition pore and blocking cytochrome-c release, thereby inhibiting the apoptotic cascade [210,288]. It is also capable of inhibiting the NLRP3 inflammasome [210,219,289]. This mechanism delivers systemic protection to the kidneys and central nervous system even in the absence of a competent microbiome, effectively bypassing the collaborative blockade. In the context of cognitive CDS, TUDCA mitigates neuroinflammation by inhibiting microglia and astrocyte activation and reducing amyloid-beta (Aβ) plaque load [210,290].

This pharmacological approach is supported by translational data from human primary biliary cirrhosis studies, where UDCA has been established as the long-term standard of care for delaying histological progression and improving clinical outcomes [291]. In veterinary medicine, the clinical management of vascular anomalies often involves metabolic stabilization prior to or in lieu of surgery. Konstantinidis et al. (2023) emphasize that medical management aims to reduce the neurotoxic effects of the bypass, but long-term stabilization may also benefit from the neuroprotective effects of TUDCA, which protects the BBB and mitochondrial membranes against the persistent oxidative stress characteristic of shunting-induced HE [28,219].

Clinical utility in dogs has been further clarified by Lee et al. (2025). In a year-long study of 60 dogs, a combination of UDCA, S-adenosyl methionine, and silymarin produced significantly superior outcomes compared to UDCA monotherapy, achieving a marked reduction in liver enzyme levels (GGT, ALP, ALT, AST) and a significant 72% decrease in gallbladder sludge percentage compared to only 28% in the UDCA monotherapy group (*p* < 0.05). This highlights the necessity of multimodal support in managing anatomical gallbladder failures [101].

#### 7.4.2. MCBAs and Synthetic Agonists: Future Perspectives

Beyond UDCA, the future of BA therapy lies in the development of high-affinity synthetic agonists for FXR (e.g., obeticholic acid) and TGR5. These agents aim to directly recover intestinal barrier integrity and suppress systemic inflammation by reactivating the endocrine signaling pathways lost during the collaborative collapse. Human trials for these compounds are progressing well. For veterinary medicine, they offer a promising, though long-term, solution for severe cases that fail to respond to microbial engraftment [8,120,130,292].

The discovery of MCBAs synthesized via the transamidation pathway opens a new therapeutic class of postbiotics. Recent human data identified novel MCBA isomers (e.g., valine, leucine, histidine and GABA-conjugated derivatives) that exhibit unique structural signatures and potentially link gut dysbiosis to systemic GABAergic signaling [96,98]. Synthetic versions of metabolites like tryptophan-cholic acid (Trp-CA)—targeting the MRGPRE—could restore glucose homeostasis and signaling pathways without requiring bacterial engraftment [133]. This represents the ultimate goal, directly reactivating the signaling brakes lost during the collaborative collapse.

### 7.5. Strategy IV: Future Directions—SynComs and Postbiotics

The clinical approach to the gut–liver axis is undergoing a fundamental shift toward precision, encompassing both diagnostic surveillance and ecological intervention. Current translational insights now identify specific shifts in the serum pool—marked by elevations in conjugated PBAs like TCA and GCA—as reliable indicators of progressive hepatic fibrosis and hepatocellular injury [141]. Recent cohort studies have highlighted that clinical remission in bile acid diarrhea hinges more on restoring bile acid metabolism—specifically serum C4 levels—than on changes in microbial taxonomy alone [293]. Large-scale human cohort data support these findings, where specific elevations in GCA and TCA—and their ratios—function as distinct metabolic signatures correlating with fibrosis severity across various etiologies [294]. These elevations often precede manifest hyperbilirubinemia, offering a non-invasive window into “silent” hepatobiliary damage that complicates the interpretation of routine TBA values [224,295].

#### 7.5.1. Precision Ecology: Transitioning to Synthetic Consortia (SynComs)

To address these specific metabolic deficits, veterinary gastroenterology is moving away from poorly defined fecal transplants toward precision ecology via SynComs. To circumvent the variability of donor material, SynComs employ lab-assembled bacterial mixtures—incorporating targeted strains such as *P. hiranonis*, *Blautia*, and *Faecalibacterium*—to re-establish critical pathways like 7α-dehydroxylation [155]. This shift enables rational design. Unlike the inconsistent nature of undefined fecal slurries, engineered SynComs consist of selected guilds optimized for specific metabolic outputs [296,297]. The transition to defined SynComs is also a safety requirement. This transition is also driven by safety concerns, as recent screenings of commercial veterinary probiotics have revealed a high prevalence of transferable antimicrobial resistance genes [264,298,299]. Identical risks apply to fecal microbiota transplantation (FMT), where the transfer of donor-derived resistance genes can lead to the long-term expansion of the recipient’s resistome [265,271,300].

While successful human clinical trials with defined bacterial spores have established the groundwork [301], veterinary medicine has recently produced its own proof-of-concept evidence. A prospective pilot study by Doshier et al. (2026) represents a significant milestone, evaluating a probiotic composed of fermentation products. It should be noted, however, that this study utilized fermentation products rather than live organisms, and baseline *P. hiranonis* levels were not quantified. Administration of this consortium led to clinical improvement in more than 70% of dogs with chronic diarrhea and stabilized fecal microbiome structure [302].

The necessity of *P. hiranonis* within these consortia has been further validated mechanistically. In a porcine “IsoLoop” model, Bayne et al. (2025) demonstrated that inoculation with *P. hiranonis* alone was sufficient to drive a functional convergence of the metagenome toward a healthy state. The bacterium activated epithelial repair pathways and restituted SBA concentrations that were otherwise absent [303].

A key illustration of this precision is the application of *Clostridium butyricum* MIYAIRI 588 strain. Recent trials confirmed that this specific strain, whether administered as a mono-therapeutic or as part of a minimal consortium, effectively modulates the gut–liver axis. This modulation occurs by enhancing Treg differentiation and suppressing hepatic lipid accumulation, thereby replicating the benefits associated with more complex FMT [304,305,306].

These defined communities provide a standardized, reproducible therapeutic modality, eliminating the inherent safety risks of fecal microbiota transplantation. Long-term engraftment of these guilds is contingent upon “ecological pairing”, as species coexistence and strain replacement dynamics are primarily determined by the recipient’s baseline microbiome structure [300]. Under this “designer synbiotic” approach, the co-administration of specific substrates provides the metabolic niche required for stable colonization [307]. Jaeger et al. (2024) demonstrated that dietary oat beta-glucan prevents steatohepatitis not simply through caloric restriction, but by specifically averting the depletion of *Lachnospiraceae* and reducing hepatic macrophage infiltration [308,309].

#### 7.5.2. Postbiotics and MCBA Analogs: Bypassing Colonization

In tandem with live therapeutics and specific substrates, postbiotics and MCBA analogs offer a strategy to bypass microbial colonization entirely. Current consensus defines postbiotics as inanimate microorganisms and/or their components that confer a physiological benefit to the host [307]. The therapeutic scope of these bioactive molecules extends to specific “gut–X” applications beyond simple metabolic replacement.

While butyrate remains foundational, its role has been redefined as a potent histone deacetylase (HDAC) inhibitor. By inhibiting HDACs, butyrate promotes histone hyperacetylation, which increases the expression of anti-inflammatory cytokines (e.g., IL-10) and reinforces the gut barrier at the epigenetic level [310,311].

Tryptophan-derived indoles, particularly indole-3-propionic acid, act as potent ligands (for the aryl hydrocarbon receptor and pregnane X receptor), reinforcing epithelial integrity. Activation of these receptors upregulates tight junction proteins, mechanically sealing the “leaky gut” associated with PLE [312,313]. The latest clinical data indicate that indole-rich postbiotics significantly reduce pruritus and clinical severity in dogs with AD, confirming that cutaneous immunity can be modulated solely through gut-derived metabolic signals [314].

Parallel to these small molecules, specific soluble proteins (p40 and p75) secreted by the *Lactobacillus rhamnosus* GG strain represent a distinct class of molecular postbiotics. These proteins activate the epidermal growth factor receptor on epithelial cells, inhibiting cytokine-induced apoptosis and maintaining barrier integrity [315]. The molecular defense extends beyond purified proteins to cell-free supernatants rich in bacteriocins and antioxidant enzymes, such as superoxide dismutase. These fractions neutralize pathogens and mitigate oxidative stress, achieving therapeutic effects independently of live bacterial engraftment [316,317]. Plant-derived bioactive compounds can further reinforce these microbial signals. Oligomeric proanthocyanidins and humic acids protect epithelial barrier integrity against pathogens (e.g., *E. coli* and *Salmonella*) and LPS-induced inflammatory damage, respectively. Other flavonoids, specifically ferulic acid and wogonin acting in synergy, alleviate cholestatic injury by mitigating bacterial translocation and enhancing BSH activity in the absence of *Clostridium* spp. By reducing oxidative stress and modulating metabolic pathways, these compounds offer a non-antibiotic alternative for barrier support and gut–liver homeostasis that avoids disrupting the resident microbiota [297,318,319,320].

Regarding mucosal repair, bacterial polyamines such as putrescine and spermidine are essential for enterocyte proliferation. Targeted supplementation with polyamine-producing strains accelerates mucosal healing in canine CE, providing a non-steroidal mechanism for barrier restitution [321,322].

#### 7.5.3. Vesicle-Based Signaling and Synthetic Agonists

A novel approach involves the use of MEVs to modulate signaling. Hong et al. (2024) demonstrated that MEVs derived from *Akkermansia muciniphila*, even in heat-killed formats, suppressed high-fat diet-induced obesity in beagles while improving insulin sensitivity and reducing visceral adiposity [323]. Proteomic mapping indicates that these vesicles translocate to the liver via the portal vein, where they modulate hepatic stellate cell activation and fibrogenesis. This confirms a direct mechanistic link in gut–liver crosstalk [11]. Under nutrient stress, certain commensals release proteolytic vesicles that modify host protease inhibitors, facilitating an adaptive communication process [324]. This signaling is not always beneficial. For instance, antibiotic stress from sub-inhibitory metronidazole has been shown to provoke the release of cytotoxic vesicles from pathobionts, intensifying the underlying inflammatory cascade [325]. These findings, alongside studies on heat-killed bacteria acting via microbe-associated molecular patterns to maintain immune vigilance, confirm that the structural integrity of the bacterium is not required to elicit a therapeutic phenotype [323]. Microbiome modulation has evolved from passive isolation to active engineering. Zu et al. (2024) introduced “nanomedicine-trained” microbiota, where gut bacteria pre-conditioned with curcumin nanocrystals secrete engineered extracellular vesicles. These “trained” EVs exhibited superior anti-inflammatory efficacy and barrier repair compared to standard FMT in ulcerative colitis models, regulating purine metabolism to decrease uric acid levels [326]. Zhou et al. (2026) further extended this principle by showing that nanoparticle-enriched, heat-inactivated lactobacilli effectively modulate mucosal immunity and NF-κB pathways. This reinforces the potential of non-viable postbiotics to achieve targeted immunomodulation while bypassing the safety risks of live microorganisms. As postbiotics, these inanimate microbial components offer a practical solution to the safety and storage challenges often associated with live bacterial treatments [327,328,329,330]. The critical signaling resides within the vesicle or cell wall components [12,331,332,333]. Scaling the isolation, purification and standardization of vesicle cargo remains a primary barrier to clinical translation [137,331,334]. Successful standardization would position these formulations as a more reliable alternative to live biotherapeutics, offering the logistical advantages of extended stability and more consistent dosing [328].

**Figure 5:**
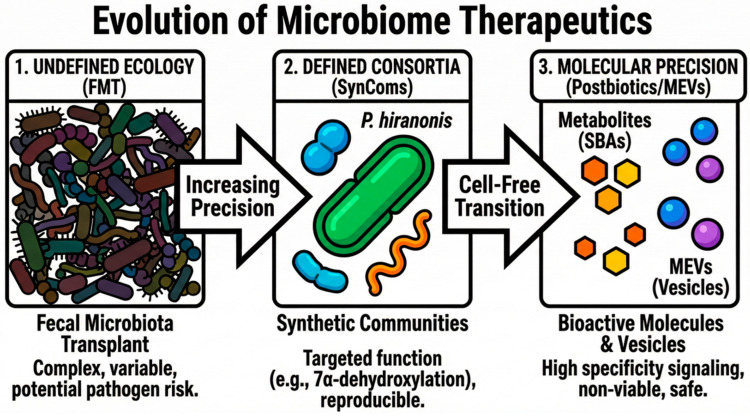
The evolution of microbiome therapeutics from undefined ecologies to molecular precision. Defined SynComs allow for targeted re-establishment by replacing specific keystone species like *P. hiranonis*. In parallel, cell-free approaches using bioactive metabolites and MEVs provide high-specificity signaling without the biological risks associated with viable organisms. Abbreviations: C4, 7α-hydroxy-4-cholesten-3-one; DI, dysbiosis index; FMT, fecal microbiota transplantation; P/S, primary-to-secondary bile acid ratio; SynComs, synthetic microbial consortia; TBA, total bile acids; Tx, treatment.

Synthetic analogs of beneficial metabolites, including Trp-CA and specific UDCA isoforms, directly engage host receptors (TGR5, FXR, MRGPRE) to resolve inflammation [133]. By delivering a consistent molecular signal directly to the host, this approach effectively decouples clinical success from the uncertainties of bacterial engraftment [155]. Figure 5 summarizes this evolution in microbiome therapeutics, shifting from undefined ecologies toward molecular precision, while Table 3 outlines these current and emerging strategies, including their mechanisms and clinical indications.

## 8. Synthesis and Future Directions: Perspectives on Biological Integration

### 8.1. Conceptual Synthesis: The Collaborative Axis in Transition

The evidence synthesized in this review confirms a necessary shift in how BAs are conceptualized in veterinary medicine. Moving beyond their classical role as digestive detergents [1,61,335], they are now recognized as systemic signaling molecules managed by a collaborative metabolome between the host and the gut microbiome [10,15].

We propose that the disruption of this axis—which we term the collaborative collapse—represents a central pathogenic event in a wide spectrum of diseases. This collapse is not solely an effect of intestinal inflammation but a driver of it, creating a state of TGR5/FXR signaling starvation. By integrating recent metabolomic data, we can move toward a more structured understanding of these dysmetabolic states, categorizing them based on their primary physiological failure.

### 8.2. A Proposed Functional Classification of Dysmetabolism

Instead of a generic diagnosis of dysbiosis, we propose a classification scheme that stratifies patients based on the distinct pathogenic mechanisms identified in current research:

Profile 1: functional microbial failure (SBA depletion). This characterizes the microbial collapse seen in primary GI diseases (CE, PLE, EPI). The pathognomonic feature is the loss of the keystone converter, *P. hiranonis*, leading to the extinction of anti-inflammatory SBAs and the consequent failure of the TGR5-GLP-1 axis [19,26,91]. This profile extends to systemic conditions like heart failure and CKD, where the metabolic insufficiency fuels systemic inflammation [15,37,40].

Profile 2: host-driven biliary excess (PBA spillover). This reflects a primary defect in hepatobiliary containment or transport (BTD, GBM, BAD). Here, the microbial metabolic apparatus may remain intact, but it is overwhelmed by an unregulated influx (spillover) of PBAs into the colon, inducing secretory diarrhea and mucosal irritation independent of microbial status [31,94]. Table 4 compares the diagnostic markers and therapeutic objectives for these divergent pathological profiles.

### 8.3. Refined Diagnostic Strategy: Integrating the Multi-Matrix Profiling Approach

The evidence synthesized in this review mandates a transition from isolated laboratory tests to an integrated, multi-matrix diagnostic strategy. Beyond the measurement of total concentrations, the true state of the collaborative metabolome is revealed through the synchronized analysis of serum and fecal profiles. This approach allows clinicians to distinguish between markers of host-driven clearance (the “upward” liver readout) and the systemic flux of microbially modified metabolites (the “outward” gut readout), providing a molecular basis for differentiating primary GI failure from hepatobiliary spillover [17,64,224].

#### 8.3.1. Serum and Plasma Profiling: The Systemic Signaling Readout

Modern diagnostics utilize LC-MS/MS to quantify up to 15–20 individual BA species in canine and feline serum, establishing that serum and plasma concentrations are physiologically comparable and provide a high-resolution view of the systemic BA pool [64]. While serum TBA pairs (12 h fasting and 2 h PP) remain essential for evaluating hepatic extraction and vascular integrity [18,76,79], targeted profiling reveals more granular shifts. In healthy dogs, the serum pool is characterized by a dominance of conjugated PBAs (e.g., TCA, TCDCA), with SBAs occurring at significantly lower concentrations than in feces [64,164]. Yet, in pathological states, specific serum profiles serve as early-warning markers: elevated serum TCA and GCA concentrations are now recognized as sensitive indicators of progressive hepatic fibrosis and cholestatic injury even before observable hyperbilirubinemia occurs [31,141]. Moreover, in feline medicine, the serum profile is uniquely sensitive to nutritional status; taurine depletion leads to a marked increase in unconjugated BAs in serum, reflecting a failure of hepatic conjugation capacity that precedes clinical signs of deficiency [59]. From a systemic perspective, the serum P/S ratio provides a clinical window into the “neuro-metabolic shield” where a decline in serum SBAs is linked to cognitive dysfunction and depression-like phenotypes driven by gut dysbiosis [156].

#### 8.3.2. Fecal BA Profiling: Interpreting the Intraluminal Fingerprint

Fecal profiling serves as the primary readout of the microbial refinery, quantifying the considerable structural complexity linked to intestinal morphology and residence time [147]. Unlike serum, where conjugated species dominate, the healthy fecal pool is over 90% unconjugated SBAs (DCA, LCA). The synchronized measurement of the fecal P/S ratio alongside the serum profile identifies the microbial collapse even when serum TBA values remain within reference intervals [25,144,146]. This contrast is instructive: CE and PLE patients typically exhibit a primary-dominant fecal profile (SBA deficiency) while maintaining relatively stable serum TBA pairs, whereas spillover cases (BTD, GBM) show PBA accumulation in both matrices [30,31,94].

#### 8.3.3. The DI as a Functional Proxy

The DI provides a rapid, qPCR-based assessment of the microbial guild structure, specifically predicting the loss of *P. hiranonis*. Depletion of this keystone species below 10^5^ gene copies/g dry feces serves as a reliable predictor of the transition from a healthy fecal SBA profile to the primary-dominant state seen in microbial collapse [19,25,128,148,149]. The DI thus acts as a screening tool to identify candidates for FMT or targeted dietary modulation before systemic signaling deficits lead to irreversible organ damage [149,151].

The future of diagnostics lies in the integration of BAs with other gut health markers. Rey et al. (2025) emphasized that combining BA profiling with the measurement of fatty acids and Aas provides a comprehensive readout of intestinal functionality and microbiome activity. For example, a concurrent reduction in SCFAs and alterations in tryptophan-derived metabolites serve as indicators of metabolic fragility before clinical organ failure occurs [170].

By integrating these matrices into a comprehensive diagnostic consideration, clinicians can begin to identify mechanism-based patterns that guide precision intervention. For instance, a profile characterized by healthy serum TBA but a primary-dominant fecal P/S ratio strongly suggests microbial collapse pointing towards a need for microbial restitution.

**Figure 6:**
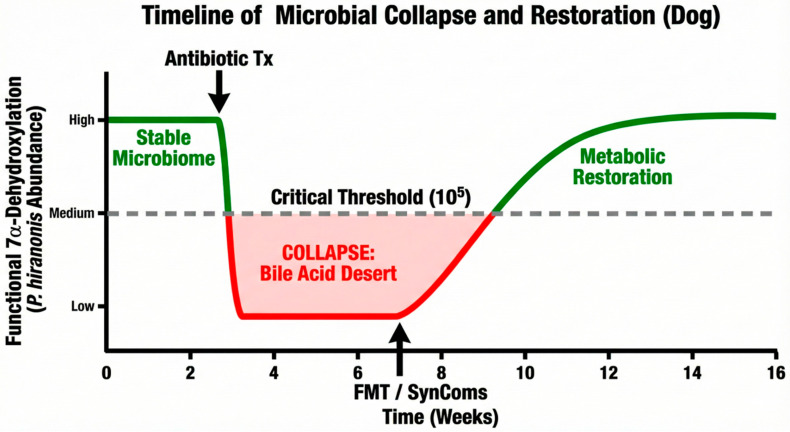
Proposed functional diagnostic algorithm for differentiating bile acid dysmetabolism profiles. This approach integrates multi-matrix profiling, combining fecal markers (Primary-to-Secondary ratio, Dysbiosis Index, and quantification of *Peptacetobacter hiranonis*) with serum biomarkers (C4, postprandial total bile acid). By reliably distinguishing between functional microbial failure (“Microbial collapse”) and host-driven containment failure (“Hepatobiliary Spillover”), this algorithm guides the selection of mechanism-based therapies, such as microbial recovery (FMT or synthetic microbial consortia SynComs) or metabolic sequestration. Abbreviations: MEVs, microbial extracellular vesicles; SBA, secondary bile acid; SynComs, synthetic microbial consortia.

In cases where elevated serum TBA is accompanied by a PBA-rich fecal profile despite normal *P. hiranonis* abundance, the clinical picture suggests hepatobiliary spillover as the primary pathology, effectively ruling out microbial failure and indicating that metabolic sequestration should be the therapeutic priority. When exceptionally high PP serum TBA occurs alongside a healthy fecal P/S ratio, the diagnostic focus must shift toward investigating potential vascular bypass. Integrating these markers into a multi-layered diagnostic framework enables a more precise clinical approach, allowing practitioners to distinguish between the need for restoring the microbial guild and the requirement for stabilizing host vascular or hepatic functions (Figure 6) [29,141,186,224].

### 8.4. Uncharted Territory: The Fifth Mechanism and Neuro-Metabolic Control

The identification of the fifth mechanism—the microbial conjugation of BAs with AAs like tryptophan and phenylalanine—expands our understanding of host–microbe communication. These MCBAs act as specific ligands for host receptors (e.g., MRGPRE), directly linking gut status to systemic glucose homeostasis and insulin sensitivity [98,133].

The recent characterization of the TGR5-BDNF pathway further extends this influence to the gut–brain axis, confirming the microbiome is a direct regulator of cognitive plasticity and neuroprotection. The loss of these neuroactive metabolites during collapse provides a molecular rationale for the “gut–brain” comorbidities—anxiety, epilepsy, and cognitive dysfunction—seen in chronic enteropathy patients [206].

The identification of MEVs and distinct postbiotic markers, such as indoles and polyamines, is transforming current therapeutic strategies. This evidence indicates a transition from cellular methods, such as live bacterial transplants, toward molecular interventions. Doshier et al. (2026) demonstrated the clinical efficacy of the *P. hiranonis*/*M. funiformis*/*E. faecium* consortium in dogs, establishing SynComs as a practical therapeutic option [302]. Parallel to these live consortia, synthetic MCBA analogs and vesicle-mediated delivery systems (including those capable of portal translocation) offer a way to calibrate host metabolic functions with pharmacological precision [11,12,296].

### 8.5. Breed-Specific Baselines and the “Hidden Pool”

Recent in vitro data from the CANIM-ARCOL model have emphasized that intestinal TT, governed largely by host body size, reshapes the BAP. Specifically, large-breed dog models exhibit significantly higher SCFA and gas production (H_2_, CO_2_) compared to small-breed counterparts, reflecting a developed fermentative process that naturally accelerates the conversion of PBAs to SBAs [106]. This relationship is supported by the observation that BA residence time is a primary determinant of the structural complexity found in vertebrate feces, with longer transit favoring more extensive microbial modification [60,61,147].

Moreover, murine models have recently uncovered stark sex-dependent differences in the portal BA composition, with males exhibiting three times higher concentrations of microbially oxidized oxo-BAs compared to females [336]. This sexual dimorphism in microbe-mediated BA transformation may necessitate gender-specific diagnostic thresholds in future veterinary protocols.

Establishing size-specific reference intervals for the P/S ratio and DI is essential to avoid misinterpreting physiological variation as pathological failure. The roles of iso- and oxo-BAs, which may constitute up to 30% of the fecal pool, remain largely unexplored in dogs and cats and represent a pressing area for future immunological research [125].

## 9. Conclusions

The shift toward mechanism-based classification marks a significant maturation of veterinary gastroenterology. This review establishes that BA dysmetabolism is a central, bi-directional driver of pathology, linking gut health to systemic homeostasis, moving beyond its traditional characterization as a downstream consequence of inflammation. Viewing microbial collapse as a systemic endocrine failure, characterized by impaired TGR5 and FXR signaling, allows clinicians to move beyond non-specific immunosuppression toward precision modulation of the host–microbe axis.

Integrating functional diagnostics, such as the DI and targeted fecal BA profiling, enables the differentiation between primary microbial failure and host-driven hepatobiliary spillover. This distinction determines whether therapy should prioritize microbial restoration or the sequestration of toxic metabolites. Beyond intestinal symptoms, the elucidation of “gut–X” axes—reflecting the biological principle of organ crosstalk—highlights that metabolic restitution is essential for preserving renal, cardiac, cutaneous, skeletal and neurological integrity. The clinical application of defined SynComs and the validation of MEVs signal a move toward precision ecology, offering standardized alternatives to the inherent variability of donor-derived material for managing complex, multisystemic diseases in veterinary patients.

## Figures and Tables

**Table 1:** Functional classification of the collaborative bile acid metabolome in companion animals. The table categorizes bile acids based on their origin, conjugation status, and physiological impact, highlighting the distinction between host-derived and microbially generated signaling molecules. Abbreviations: *bai*, bile acid-inducible operon; BSH, bile salt hydrolase; CA, cholic acid; CDCA, chenodeoxycholic acid; CE, chronic enteropathy; DCA, deoxycholic acid; ER, endoplasmic reticulum; FMT, fecal microbiota transplantation; FXR, farnesoid X receptor; GCA, glycocholic acid; GLP-1, glucagon-like peptide-1; LCA, lithocholic acid; MCBAs, microbially conjugated bile acids; MRGPRE, Mas-related G protein-coupled receptor member E; *P. hiranonis*, *Peptacetobacter hiranonis*; Phe-CA, phenylalanine-cholic acid; PLE, protein-losing enteropathy; TCA, taurocholic acid; TCDCA, taurochenodeoxycholic acid; TGR5, transmembrane G protein-coupled receptor 5; Trp-CA, tryptophan-cholic acid; TUDCA, tauroursodeoxycholic acid; Tyr-CA, tyrosine-cholic acid; T-β-MCA, tauro-beta-muricholic acid; UDCA, ursodeoxycholic acid.

Functional Class	Key Representatives (Canine/Feline)	Origin/Enzymatic Driver	Primary Biological Role/Mechanism	Pathological Context
**Primary Conjugated (Host)**	TCA, TCDCA, GCA, T-β-MCA	Liver/host conjugation (taurine > glycine)	Digestive: lipid emulsification (micelle formation) Signaling: FXR activation (synthesis brake)	Hepatobiliary spillover: accumulation drives secretory diarrhea and mucosal permeability
**Primary Unconjugated (Transitional)**	CA, CDCA	Proximal gut/bacterial BSH (e.g., *Clostridium*, *Lactobacillus*)	Intermediate: substrate for 7α-dehydroxylation. low solubility; potential cytotoxicity if accumulated	BSH-active Dysbiosis: premature deconjugation causes fat malabsorption and mucosal irritation
**Secondary (Microbial Signals)**	DCA, LCA, UDCA	Distal gut/7α-dehydroxylation (*P. hiranonis*, *bai* operon)	Immuno-metabolic: potent TGR5 agonists (anti-inflammatory brake, GLP-1 secretion)	Microbial collapse: depletion leads to “signaling starvation” and chronic inflammation (CE, PLE)
**Tertiary/Cytoprotective**	UDCA, TUDCA	Hepato-biliary recycling/epimerization	Cytoprotection: membrane stabilization, ER stress reduction, anti-apoptotic.	Cholestasis/renal fibrosis: deficiency exacerbates cellular injury in liver and kidney
**Novel Conjugates (MCBAs)**	Trp-CA, Phe-CA, Tyr-CA	Gut microbiota/transamidation (altered BSH activity)	Systemic signaling: specific agonists for novel receptors (e.g., MRGPRE); modulate glucose/insulin	Metabolic syndrome: potential loss contributes to systemic metabolic dysregulation

**Table 2:** The Gut–X axis network: key metabolic mediators and clinical consequences in companion animals. The upward (↑) and downward (↓) arrows indicate an increase or decrease in the respective parameters. Abbreviations: TMAO, trimethylamine N-oxide; MEV, microbial extracellular vesicle; MMVD, myxomatous mitral valve disease; UDCA, ursodeoxycholic acid; CKD, chronic kidney disease; IRIS, International Renal Interest Society; BA, bile acid; BDNF, brain-derived neurotrophic factor; GABA, gamma-aminobutyric acid; BBB, blood–brain barrier; CDS, cognitive dysfunction syndrome; SCFA, short-chain fatty acid; Treg, regulatory T cell; TCA, taurocholic acid; GCA, glycocholic acid; LPS, lipopolysaccharide; FXR, farnesoid X receptor; MASLD, metabolic dysfunction-associated steatotic liver disease.

Target Axis	Key Metabolites/Mediators	Pathological Mechanism	Clinical Consequence
**Gut–Heart**	↑ TMAO, MEVs	Pro-inflammatory signaling, myocardial fibrosis, remodeling	Progression of MMVD, left atrial enlargement
**Gut–Kidney**	↓ UDCA, ↑ Uremic Toxins (Indoxyl sulfate, p-cresol)	Loss of cytoprotection, direct nephrotoxicity, tubulointerstitial fibrosis	Progression of CKD (IRIS Stages 2–4), renal fibrosis
**Gut–Brain**	↓ Secondary BAs, ↓ BDNF	Neuroinflammation, altered GABA/glutamate balance, BBB permeability	Epilepsy, cognitive dysfunction (CDS), hepatic encephalopathy
**Gut–Skin**	↓ SCFA (butyrate), altered BA pool	Impaired Treg differentiation, loss of immune tolerance, systemic pruritus	Atopic dermatitis, chronic pruritus
**Gut–Liver**	↑ Primary BAs (TCA, GCA), LPS translocation	Failure of FXR feedback, stellate cell activation, inflammation	MASLD, hepatocellular injury, fibrosis

**Table 3:** Therapeutic strategies for restoring the collaborative metabolome. Interventions are grouped by their clinical objective: metabolic guild restoration (e.g., FMT, SynComs) or the management of metabolic toxicity (e.g., BAS, UDCA). Abbreviations: FMT, fecal microbiota transplantation; BAS, bile acid sequestrants; UDCA, ursodeoxycholic acid; TUDCA, tauroursodeoxycholic acid; SynComs, synthetic Microbial consortia; MEVs, microbial extracellular vesicles; *P. hiranonis*, *Peptacetobacter hiranonis*; SBA, secondary bile acid; PBA, primary bile acid; CE, chronic enteropathy; PLE, protein-losing enteropathy; *C. difficile*, *Clostridioides difficile*; BAD, bile acid diarrhea; GBM, gallbladder mucocele; HE, hepatic encephalopathy; CDS, cognitive dysfunction syndrome; DI, dysbiosis index; ER, endoplasmic reticulum; TGR5, transmembrane G protein-coupled receptor 5; FXR, farnesoid X receptor; MRGPRE, Mas-related G protein-coupled receptor member E; Trp-CA, tryptophan-conjugated cholic acid; IBD, inflammatory bowel disease.

Strategy/Agent	Mechanism of Action	Target Condition	Key Considerations/Dosing Note
**FMT**	Restores functional guild (*P. hiranonis*), normalizes SBA production	CE, PLE, refractory *C. difficile*	Donor screening (DI < 0) is critical; repeated dosing often required
**BAS (cholestyramine, colesevelam)**	Binds excess luminal PBAs, reduces secretory toxicity	BAD, hepatobiliary spillover	Monitor for malabsorption of fat-soluble vitamins; separate from other meds
**UDCA/TUDCA**	Cytoprotection, membrane stabilization, ER stress reduction	Cholestasis, GBM, neuroprotection (HE/CDS), renal fibrosis	Hydrophilic tertiary BA; does not correct dysbiosis but bypasses toxicity
**Prebiotics (psyllium)**	Mechanical sequestration + prebiotic support for fermenters	Chronic diarrhea, barrier support	Induces hepatic synthesis overshoot; stabilizes fecal consistency.
**SynComs (Future)**	Precision engraftment of *P. hiranonis* and supporters	Targeted restoration without pathogen risk	Emerging therapy; aimed at defined metabolic output (7α-dehydroxylation)
**MCBAs/Synthetic Agonists**	Direct receptor activation (TGR5, FXR, MRGPRE), bypassing dysbiosis	Metabolic syndrome, barrier dysfunction, systemic inflammation	Highly specific signaling molecules (e.g., Trp-CA); decouples effect from colonization
**MEVs/Postbiotics**	Bioactive cargo delivery (proteins, mRNA), barrier reinforcement, immunomodulation	IBD, barrier dysfunction, systemic inflammation (Gut-X axis)	Non-living therapeutic; high stability; standardization challenges remain

**Table 4:** Comparison of the two primary pathological profiles of bile acid dysmetabolism. Abbreviations: BA, bile acid; TGR5, Takeda G protein-coupled receptor 5; FXR, farnesoid X receptor; PBA, primary bile acid; SBA, secondary bile acid; P/S, primary-to-secondary ratio; TBA, total bile acid; C4, 7α-hydroxy-4-cholesten-3-one; PP, postprandial; DI, dysbiosis index; *P. hiranonis*, *Peptacetobacter hiranonis*; CE, chronic enteropathy; PLE, protein-losing enteropathy; EPI, exocrine pancreatic insufficiency; MMVD, myxomatous mitral valve disease; BAD, bile acid diarrhea; PSS, portosystemic shunt; FMT, fecal microbiota transplantation; SynComs, synthetic microbial consortia; BAS, bile acid sequestrants.

Feature	Profile 1: Microbial Collapse (Functional Failure)	Profile 2: Hepatobiliary Spillover (Containment Failure)
**Primary Defect**	Loss of 7α-dehydroxylating guild (*P. hiranonis*)	Host-driven secretory excess or transport failure
**Key Mechanism**	“Signaling starvation” (lack of TGR5/FXR ligands)	“Secretory toxicity” (smotic/detergent effect of PBAs)
**Fecal BA Profile (P/S Ratio)**	Inverted (high P/S): dominance of PBAs, absence of SBAs	Mixed or primary-rich: high total fecal BA excretion, variable P/S
**Serum Biomarkers**	Often normal TBA; low circulating SBAs	Elevated C4 (synthesis marker); elevated PP TBA
**Microbial Status (DI)**	Dysbiotic: low *P. hiranonis* (<10^5^), low diversity	Often eubiotic: normal *P. hiranonis*, or concurrent dysbiosis
**Associated Diseases**	CE, PLE, EPI, MMVD, renal fibrosis	BAD, Gallbladder Mucocele, PSS
**Therapeutic Goal**	Restoration: repopulate the guild (FMT, SynComs)	Sequestration: bind excess metabolites (BAS)

## Data Availability

No new data were created or analyzed in this study. Data sharing is not applicable to this article.

## Abbreviations

The following abbreviations are used in this manuscript:

AA	Amino acid
AD	Atopic dermatitis
ALP	Alkaline phosphatase
ALT	Alanine transaminase
ARE	Antibiotic-responsive enteropathy
ASBT	Apical sodium-dependent bile acid transporter
AST	Aspartate transaminase
AUC	Area under the curve
BA	Bile acid
BAD	Bile acid diarrhea
*bai*	Bile acid-inducible (operon)
BAP	Bile acid profile
BARF	Biologically appropriate raw food
BAS	Bile acid sequestrants
BBB	Blood–brain barrier
BDNF	Brain-derived neurotrophic factor
BSH	Bile salt hydrolase
BTD	Biliary tract disease
C4	7α-hydroxy-4-cholesten-3-one
CA	Cholic acid
CADESI-04	Canine Atopic Dermatitis Extent and Severity Index, 4th iteration
cAMP	Cyclic adenosine monophosphate
CCK	Cholecystokinin
CDCA	Chenodeoxycholic acid
CDS	Cognitive dysfunction syndrome
CE	Chronic enteropathy
CFTR	Cystic fibrosis transmembrane conductance regulator
CIBDAI	Canine IBD activity index
CKD	Chronic kidney disease
CRP	C-reactive protein
CYP27A1	Sterol 27-hydroxylase
CYP7A1	Cholesterol 7α-hydroxylase
DCA	Deoxycholic acid
DI	Dysbiosis index
DM	Diabetes mellitus
EHC	Enterohepatic circulation
EPI	Exocrine pancreatic insufficiency
FGF	Fibroblast growth factor
FMT	Fecal microbiota transplantation
FoxP3	Forkhead box P3
FXR	Farnesoid X receptor
GABA	Gamma-aminobutyric acid
GBM	Gallbladder mucocele
GCA	Glycocholic acid
GGT	Gamma-glutamyl transferase
GLP-1	Glucagon-like peptide-1
HDAC	Histone deacetylase
HE	Hepatic encephalopathy
IBD	Inflammatory bowel disease
IL	Interleukin
ILC	Innate lymphoid cell
IS	Indoxyl sulfate
LCA	Lithocholic acid
LCFA	Long-chain fatty acid
LC-MS/MS	Liquid chromatography–tandem mass spectrometry
MASLD	Metabolic dysfunction-associated steatotic liver disease
MCBA	Microbially conjugated bile acid
MEV	Microbial extracellular vesicle
MMVD	Myxomatous mitral valve disease
MRGPRE	Mas-related G protein-coupled receptor member E
MrMRE	Microbiota-related modulation-responsive enteropathy
MVD	Microvascular dysplasia
NETs	Neutrophil extracellular traps
NF-κB	Nuclear factor kappa-light-chain-enhancer of activated B cells
NLRP3	NOD-, LRR-, and pyrin domain-containing protein 3
PAMP	Pathogen-associated molecular pattern
PBA	Primary bile acid
PKA	Protein kinase A
PLE	Protein-losing enteropathy
PLN	Protein-losing nephropathy
PP	Postprandial
P/S	Primary-to-secondary (ratio)
PSS	Portosystemic shunt
qPCR	Quantitative polymerase chain reaction
SBA	Secondary bile acid
SCFA	Short-chain fatty acid
SCWT	Soft-coated Wheaten Terrier
SeHCAT	^75^Se-homotaurocholic acid test
SIBO	Small intestinal bacterial overgrowth
SynComs	Synthetic microbial consortia
T-β-MCA	Tauro-beta-muricholic acid
TBA	Total bile acid
TCA	Taurocholic acid
TCDCA	Taurochenodeoxycholic acid
TGR5	Transmembrane G protein-coupled receptor 5
TMAO	Trimethylamine N-oxide
TNF-α	Tumor necrosis factor α
Treg	Regulatory T cell
Trp-CA	Tryptophan-cholic acid
TT	Transit time
TUDCA	Tauroursodeoxycholic acid
UDCA	Ursodeoxycholic acid
